# The mammalian CTLH complex is an E3 ubiquitin ligase that targets its subunit muskelin for degradation

**DOI:** 10.1038/s41598-019-46279-5

**Published:** 2019-07-08

**Authors:** Matthew E. R. Maitland, Gabriel Onea, Christopher A. Chiasson, Xu Wang, Jun Ma, Sarah E. Moor, Kathryn R. Barber, Gilles A. Lajoie, Gary S. Shaw, Caroline Schild-Poulter

**Affiliations:** 1 Robarts Research Institute, Schulich School of Medicine & Dentistry, The University of Western Ontario, London, Ontario Canada; 2 Department of Biochemistry, Schulich School of Medicine & Dentistry, The University of Western Ontario, London, Ontario Canada; 3 Don Rix Protein Identification Facility, Schulich School of Medicine & Dentistry, The University of Western Ontario, London, Ontario Canada

**Keywords:** Ubiquitylation, Ubiquitylated proteins

## Abstract

The multi-subunit C-terminal to LisH (CTLH) complex is the mammalian homologue of the yeast Gid E3 ubiquitin ligase complex. In this study, we investigated the human CTLH complex and characterized its E3 ligase activity. We confirm that the complex immunoprecipitated from human cells comprises RanBPM, ARMC8 α/β, muskelin, WDR26, GID4 and the RING domain proteins RMND5A and MAEA. We find that loss of expression of individual subunits compromises the stability of other complex members and that MAEA and RMND5A protein levels are interdependent. Using *in vitro* ubiquitination assays, we demonstrate that the CTLH complex has E3 ligase activity which is dependent on RMND5A and MAEA. We report that the complex can pair with UBE2D1, UBE2D2 and UBE2D3 E2 enzymes and that recombinant RMND5A mediates K48 and K63 poly-ubiquitin chains. Finally, we show a proteasome-dependent increase in the protein levels of CTLH complex member muskelin in RMND5A KO cells. Furthermore, muskelin ubiquitination is dependent on RMND5A, suggesting that it may be a target of the complex. Overall, we further the characterization of the CTLH complex as an E3 ubiquitin ligase complex in human cells and reveal a potential autoregulation mechanism.

## Introduction

Ubiquitination, the addition of the 76 amino acid protein ubiquitin to lysine residues on target proteins, modifies protein function or turnover and regulates a wide spectrum of biological processes in development and disease^1,2^. The three-step mechanism is initiated by ATP-dependent activation of ubiquitin by E1 enzymes, conjugation to E2 enzymes and covalent attachment to a substrate recruited by an E3 ligase^3^. Subsequent elongation of ubiquitin molecules forms poly-ubiquitin chains, linked through one of its seven lysine residues or N-terminal methionine. The type of linkage dictates the fate and function of the ubiquitinated protein^2^. For example, poly-ubiquitinated chains linked through lysine 11 (K11) or 48 (K48) are known to target proteins for proteasomal degradation, while lysine 63 (K63) linked chains can trigger lysosomal degradation, endocytosis, regulate protein trafficking, or alter protein interactions^2^.

E3 ligases with Really Interesting New Gene (RING) finger domains mediate direct transfer of ubiquitin to the substrate from the E2^2,3^. The RING domains coordinate two Zn^2+^ ions in a cross braced arrangement critical for its structure, or without Zn^2+^ coordination in the case of the U-box family of E3 ligases in which case polar and charged residues substitute for the Zn^2+^ ions^4^. Approximately 600 RING finger E3 ligases exist in the human genome, underlying their importance in cell signaling regulation and the diversity of their substrates. RING E3 ligases exist as monomers, homodimers, heterodimers, or in multi-subunit complexes, such as the Cullin RING ligases, which contain subunits that function either as a scaffold, adaptor, or substrate recognition element, in addition to the RING subunit^5,6^.

The *g*lucose-*i*nduced degradation *d*eficient (Gid) complex is a multi-subunit E3 ligase in *Saccharomyces cerevisiae*. Upon replenishment of glucose to starved yeast cells, the Gid complex ubiquitinates gluconeogenic enzymes, such as fructose-1,6-bisphosphatase (Fbp1), phosphoenolpyruvate carboxykinase (Pck1) and malate dehydrogenase (Mdh2), leading to their proteasomal degradation^7,8^. These substrates are targeted by the complex via recognition of their N-terminal proline residues by Gid4 in the complex by a process termed the Pro/N-end rule pathway^8,9^. Gid4 is rapidly synthesized after glucose addition and associates with the Gid complex, thus triggering the ubiquitination of the gluconeogenic enzymes and allowing the cells to switch from gluconeogenesis to glycolysis^7^.

In the Gid complex, Gid2 and Gid9 both contain conserved RING finger domains and form a heterodimer^7,10,11^. Ubiquitin ligase activity has been demonstrated *in vitro* for yeast Gid2^7^ and its *Xenopus laevis* orthologue^12^. For Gid9, mutation of a cysteine residue in its RING domain abrogates ubiquitination and degradation of the gluconeogenic targets of the Gid complex, although activity of Gid9 in *in vitro* assays could not be demonstrated^11^.

The C-terminal to LisH (CTLH) complex is the mammalian homologue of the Gid complex^10^. It was originally identified by analysis of Ran binding protein M (RanBPM, also known as RanBP9) associated proteins in a high molecular weight fraction of HEK293 extracts, consisting of muskelin, Two Hybrid-Associated Protein 1 With RanBPM (TWA1, also known as GID8), Armadillo Repeat Containing 8 (ARMC8) isoforms α and β and two subunits containing RING domains, Macrophage Erythroblast Attacher (MAEA, also known as EMP; homologue of Gid9) and Required For Meiotic Nuclear Division 5 Homolog A (RMND5A; homologue of Gid2)^10,13,14^. The CTLH complex was named following the observation that five of the complex members contain LIS1-homology motif (LisH) and CTLH domains^10,13^.

The most well-studied complex member is RanBPM, a 90 kDa ubiquitously expressed, nucleocytoplasmic and evolutionary conserved protein implicated in a variety of cellular functions^15^. RanBPM is a regulator of multiple signaling pathways, including the ERK pathway, Transforming growth factor-β (TGF-β), histone deacetylase 6 (HDAC6) activity and the Ataxia Telangiectasia Mutated (ATM)-dependent DNA damage response^15–17^. Other CTLH subunits, such as muskelin, have been implicated in intracellular protein trafficking, microtubule dynamics and cell adhesion, whereas MAEA has been found to regulate erythroblast and macrophage maturation^18–21^. Few studies so far have evaluated the biological function of the complex in mammalian cells^15,22,23^. While most subunits of the complex are conserved within eukaryotic lineages and the RING domains in MAEA and RMND5A share high levels of conservation with their yeast counterparts^10^, the composition, activity and function of the complex remain to be characterized.

In this study, we further the characterization of the CTLH complex and investigate its E3 ubiquitin ligase activity in human cells. We find that WD repeat-containing protein 26 (WDR26) and GID4 are components of the CTLH complex and that complex members are differentially distributed within nuclear and cytoplasmic compartments. Through analysis of knockout cell lines of CTLH subunits, we determine that the stability of several complex members is interdependent and that, in particular, RanBPM and TWA1 are critical for complex stability. We show that the complex has E3 ligase activity and that this is dependent on both RMND5A and MAEA. Furthermore, we characterize the E2 pairings of the complex and ubiquitin linkage. Finally, we determine that the stability and ubiquitination of CTLH complex member muskelin is regulated by the complex, suggesting that this may be part of an autoregulatory mechanism.

## Results

### WDR26 and GID4 are CTLH complex members

**Figure 1:**
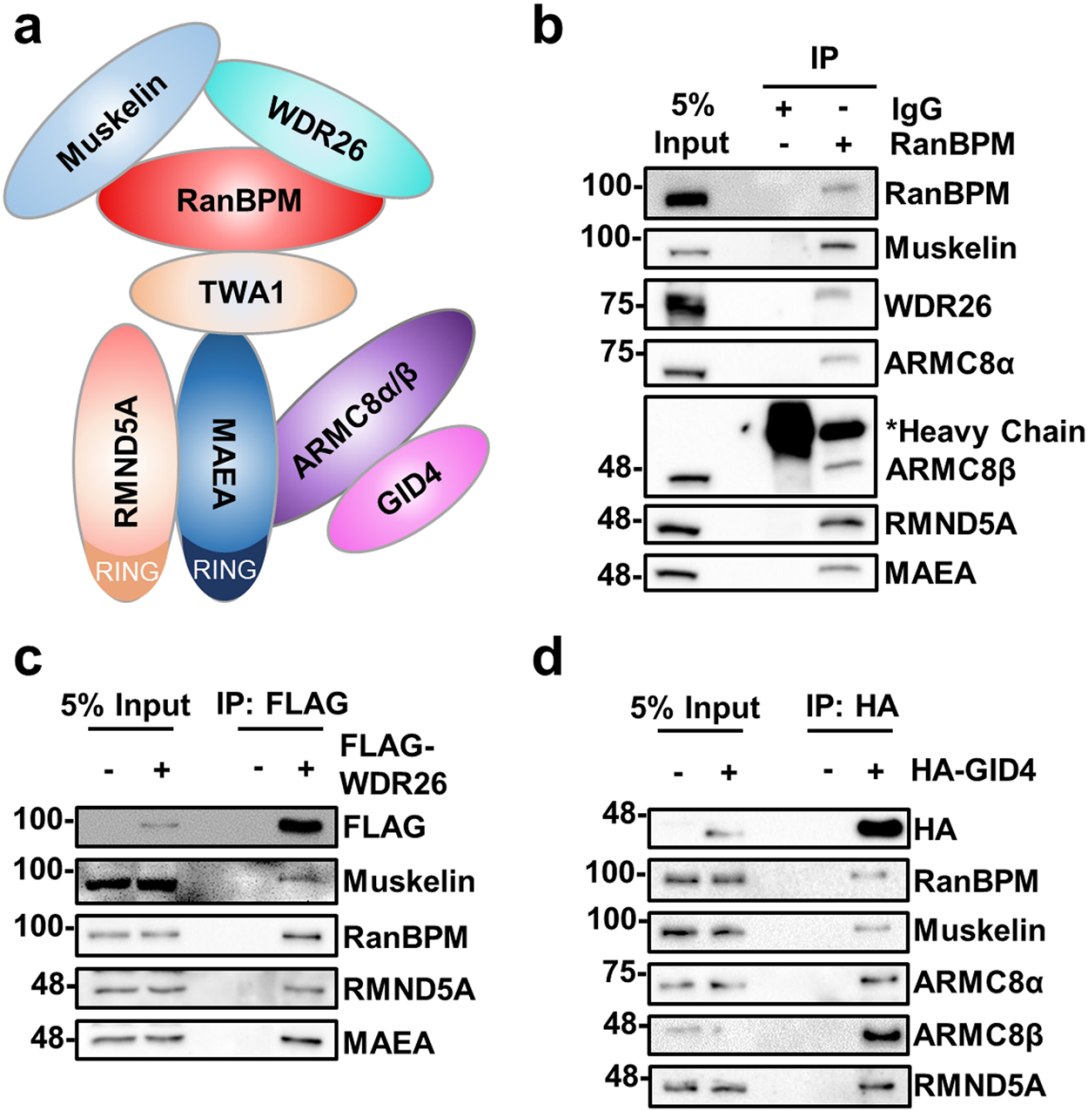
Characterization of the CTLH complex. (**a**) Schematic representation of the CTLH complex. The model is adapted from the yeast Gid complex^26^. Note that the position of muskelin in the complex has not been formally defined. (**b)** Subunits of the CTLH complex are present in RanBPM immunocomplexes. HEK293 whole cell extracts were incubated with a RanBPM antibody and immunoprecipitated. Immunoprecipitates were analyzed by Western blot with the indicated antibodies. IgG was used as a negative control. (**c**) WDR26 associates with the CTLH complex. Whole cell extracts were prepared from HeLa cells untransfected (−) or transfected with FLAG-tagged WDR26 (+). FLAG-WDR26 was immunoprecipitated with a FLAG antibody and immunoprecipitates were analyzed by Western blot with the indicated antibodies. (**d)** GID4 associates with CTLH complex. Whole cell extracts were prepared from HEK293 cells untransfected (−) or transfected with HA tagged GID4 (+). HA-GID4 was immunoprecipitated with an HA antibody and immunoprecipitates were analyzed by Western blot with the indicated antibodies.

The initial characterization of the CTLH complex determined that it was composed of 6 subunits, RanBPM, TWA1, muskelin, ARMC8 and the RING domain proteins RMND5A and MAEA (Fig. 1a)^13,14^. We confirmed the composition of the complex by immunoprecipitation of RanBPM in HEK293 cells and found that CTLH complex members remain associated with RanBPM even under stringent conditions (Fig. 1b, Supplementary Fig. 1). WD Repeat Domain 26 (WDR26) and human GID4 (also known as c17orf39), the homologues of the yeast Gid complex members Gid7 and Gid4, respectively, were not detected in the initial identification of the complex^13^. In contrast, we found that endogenous WDR26 associates with RanBPM (Fig. 1b) and that CTLH complex members co-immunoprecipitate with exogenously expressed WDR26 and GID4 (Fig. 1c,d), consistent with recent interactome studies that revealed the human proteins do associate with the complex^24,25^. Taken together, this works shows that WDR26 and GID4 are CTLH complex subunits.

**Figure 2:**
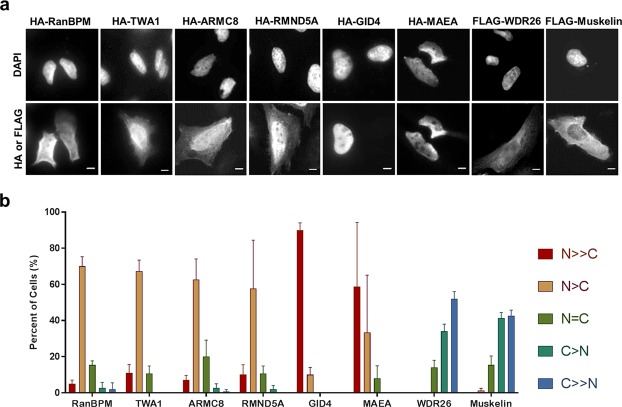
Localization of CTLH complex subunits. (**a)** Representative images of transfected HA- or FLAG-tagged CTLH complex members in HeLa cells. Scale bar: 10 μm. Hela cells fixed 24 h after transfection were incubated with an HA or FLAG antibody and then with an Alexa Fluor 488 secondary antibody. Nuclei were stained with DAPI. (**b**) Analysis of the subcellular localization of transfected CTLH complex members. Subcellular localization was scored as either, N ≫ C (completely nuclear), N > C (nuclear greater than cytoplasmic), N = C (nuclear equal to cytoplasmic), C > N (cytoplasmic greater than nuclear) or C ≫ N (completely cytoplasmic). Data represent averages from three separate experiments, each assessing approximately 50 cells. Error bars represent standard deviation (SD).

To compare the subcellular localizations of all complex members, we transfected HA or FLAG tagged constructs of RanBPM, TWA1, ARMC8, RMND5A, MAEA, muskelin, WDR26 and GID4 in HeLa cells (Fig. 2a). Consistent with a previous report^13^, RanBPM, TWA1, ARMC8 and RMND5A showed nucleocytoplasmic distribution, with a nuclear predominance, while muskelin appeared mostly cytoplasmic and MAEA nearly exclusively nuclear (Fig. 2b). Interestingly, GID4 displayed a near exclusive nuclear staining and WDR26 was primarily cytoplasmic (Fig. 2a,b). The differing subcellular localization of CTLH complex members suggests the possibility that several complexes of varying composition may co-exist in the nucleus and cytoplasm.

### Interdependence of CTLH complex subunit stability

**Figure 3:**
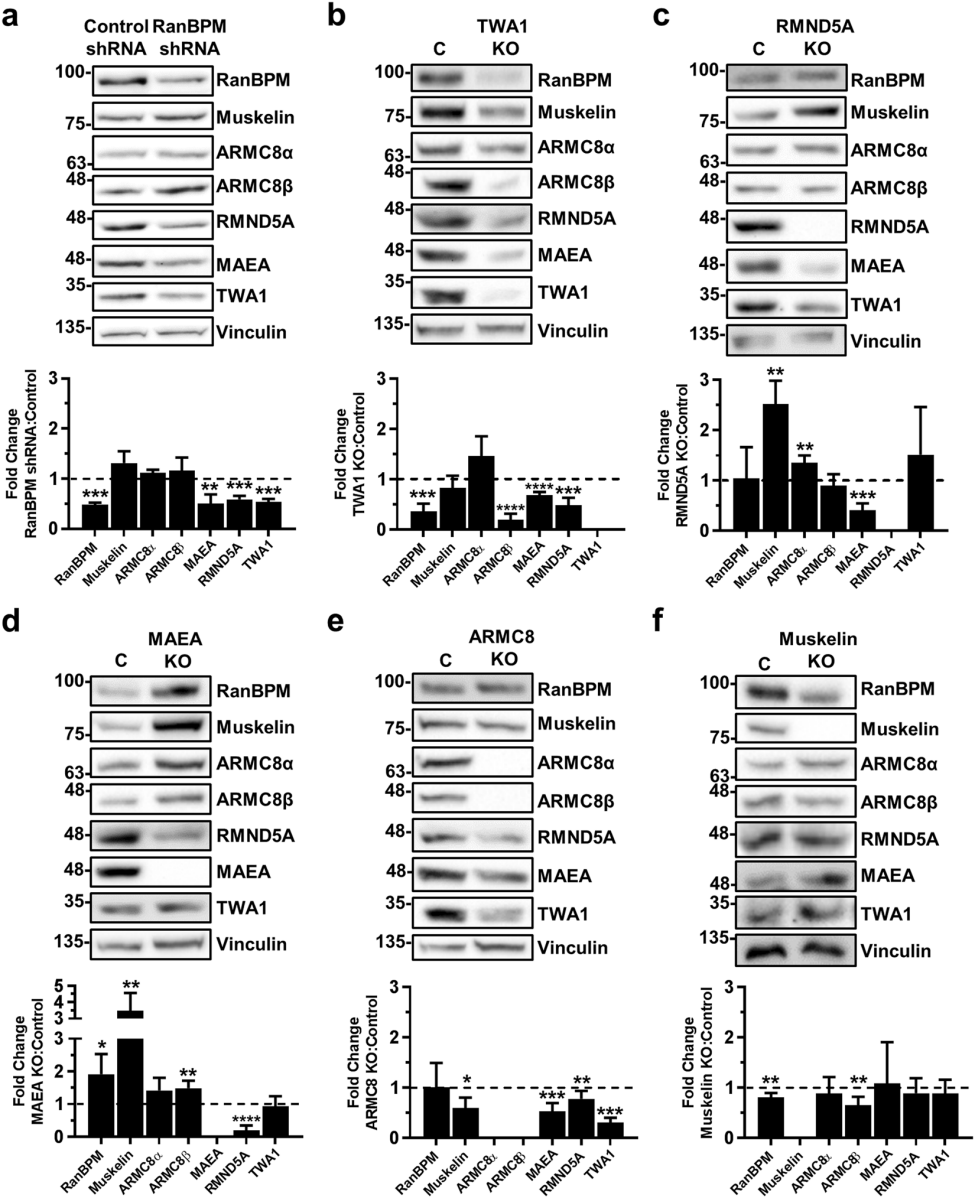
RanBPM and TWA1 are essential for complex stability. Whole cell extracts prepared from control shRNA and RanBPM shRNA HEK293 cells (**a**), or from control (labelled as C), TWA1, RMND5A, MAEA, ARMC8 and muskelin HEK293 CRISPR knockout cells (**b**–**f**) were analyzed by Western blot with antibodies to CTLH complex members, as indicated. Vinculin was used as a loading control. Quantifications are shown below each blot and protein levels are shown relative to control cells set to 1 and normalized to Vinculin levels. Data represent averages from three separate experiments, with error bars indicating SD. *P < 0.05, **P < 0.01, ***P < 0.001, ****P < 0.0001.

The RanBPM and TWA1 yeast homologues Gid1 and Gid8, respectively, form the core of the Gid complex with MAEA and RMND5A homologues (Gid9 and Gid2), which form a heterodimer (Fig. 1a). The remaining 3 subunits, Gid7, Gid5 and Gid4, the yeast homologues of WDR26, ARMC8 and GID4/C17orf39, respectively, were predicted to be located on the periphery of the complex^15,26^. If this topology is similar in the CTLH complex, RanBPM and TWA1 would be expected to be critical for complex stability. To determine how CTLH complex subunit expression influences each other, we assessed the levels of CTLH individual subunits in stable shRNA knockdown or CRISPR knockout (KO) cell lines for six of the complex members. We found that depletion of RanBPM or TWA1 strongly affected each other’s protein levels as well as that of MAEA and RMND5A (Fig. 3a,b). TWA1 knockout also had a surprising inhibitory effect on the stability of ARMC8β, while ARMC8α was not significantly changed (Fig. 3b). Interestingly, the RING dimer partners MAEA and RMND5A appeared to require each other for stability as knockout of each one individually significantly reduced the protein levels of the other (Fig. 3c,d). No other prominent change was seen in RMND5A and MAEA KO cell lines, except that both showed a significantly higher amount of muskelin (Fig. 3c,d). This was also observed in RanBPM shRNA cells, albeit to a lesser extent potentially owing to the partial downregulation of RanBPM in these cells (Fig. 3a). Knockout of ARMC8 resulted in downregulation of MAEA, RMND5A and TWA1 (Fig. 3e), suggesting that TWA1, MAEA/RMND5A and ARMC8 influence each other’s stability. Finally, the knockout of muskelin only had subtle effects, if any, on protein levels of other complex members (Fig. 3f). To determine whether these changes occurred at the mRNA level, we performed RT-qPCR analyses to evaluate whether the KO of individual subunits had an effect on the transcriptional regulation of other CTLH complex members. We did not detect any change in mRNA expression for most of the subunits tested, except for a small reduction of muskelin and MAEA mRNA in RMND5A KO cells, and a slight decrease for RMND5A mRNA in ARMC8 KO cells (Supplementary Fig. 2). In all cases, these changes were much smaller than the effects observed at the protein level (or even opposite in the case of muskelin) and therefore unlikely to account for the full extent of the effect observed at the protein level. Altogether, this substantiates that the knockout of individual complex members affects the stability of other complex members mostly at the protein level.

Finally, we used a combination of transient siRNA knockdown and subunit re-expression in knockout cell lines to confirm that these changes were not due to off-targets effects. We confirmed that siRNA downregulation of TWA1, muskelin and RMND5A recapitulated the changes observed in TWA1, muskelin and RMND5A CRISPR KO cells (Supplementary Fig. 3). Similarly, RanBPM KO cells showed similar changes in CTLH subunits as the RanBPM shRNA cells and transient re-introduction of Flag-MAEA in MAEA KO cells restored the expression of RanBPM, ARMC8 and muskelin close to the levels observed in WT cells (Supplementary Fig. 3).

### Characterization of the CTLH complex E3 ligase activity

**Figure 4:**
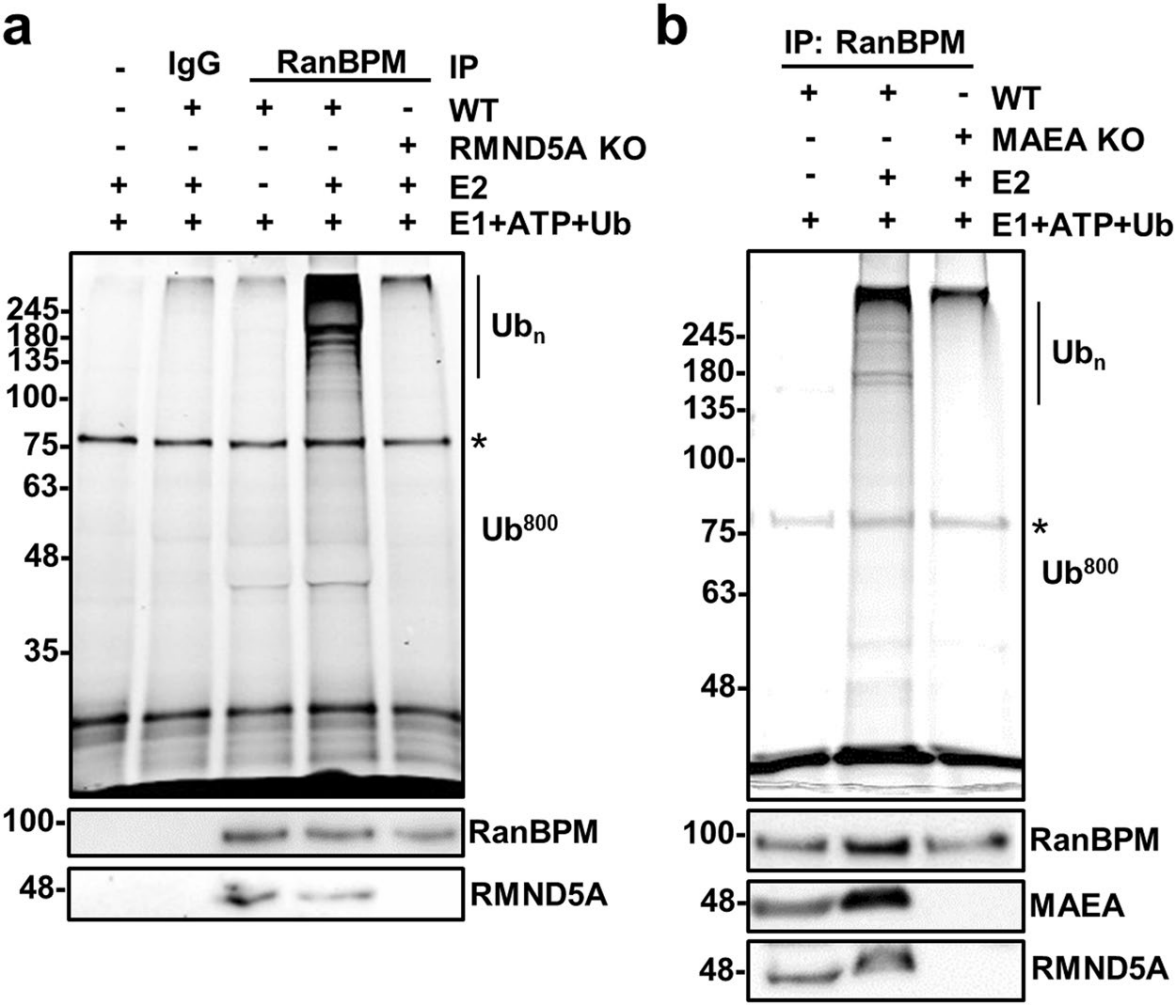
The CTLH complex has E3 ligase activity. (**a**) RanBPM immunocomplexes have E3 ligase activity that is dependent on RMND5A. Whole cell extracts prepared from wild type (WT; lanes 3–4) or RMND5A KO (lane 5) HEK293 cells were subjected to immunoprecipitation with a RanBPM antibody or an IgG control, as indicated. Immunoprecipitates were resuspended in an ubiquitination assay master mix and E2 enzyme (UBE2D2) was added (+) or omitted (−), prior to incubation at 37 °C for 30 minutes. Reactions were run on SDS-PAGE gel and fluorescein-ubiquitin was imaged directly on the gel at 800 nm. Shown below is a Western blot analyzing RanBPM and RMND5A presence in the immunoprecipitates. Asterisk indicates a non-specific band. (**b**) MAEA is required for CTLH complex activity. RanBPM was immunoprecipated from control or MAEA KO HEK293 cells and ubiquitination reactions were carried out as described above. The presence of RanBPM and MAEA in the immunoprecipitates was analyzed by Western blot. Asterisk indicates a non-specific band.

To determine whether the mammalian CTLH complex has E3 ligase activity, we conducted *in vitro* ubiquitination assays with the CTLH complex immunoprecipitated from HEK293 cells via a RanBPM antibody (as in Fig. 1b). For these assays, we supplemented the reactions with the E2 enzyme UBE2D2 (UbcH5b) because it paired with the yeast RMND5A counterpart (Gid2) in *in vitro* assays^7^ and was also identified as an interacting partner for human RMND5B, a paralog of RMND5A, in a large protein interaction screen^27^. Ubiquitination products were observed when the CTLH complex was immunoprecipitated from wild-type HEK293 cells, but not in RMND5A knockout HEK293 cells (Fig. 4a). As the complex is intact in the RMND5A KO cells (save for RMND5A, Supplementary Fig. 4), this result demonstrates that RanBPM immunocomplexes have E3 ligase activity and that it is dependent on RMND5A, a RING domain CTLH complex subunit. To understand the contributions of MAEA to the E3 ligase activity of the CTLH complex, we conducted *in vitro* ubiquitination assays with the RanBPM immunocomplexes in control and MAEA knockout HEK293 cells. As anticipated, limited E3 ligase activity was observed in the MAEA KO cells (Fig. 4b); however, consistent with the yeast Gid complex topology^26^, co-IP of RMND5A was not detected in MAEA KO cells. Therefore, the loss of activity could be attributed to the absence of RMND5A.

**Figure 5:**
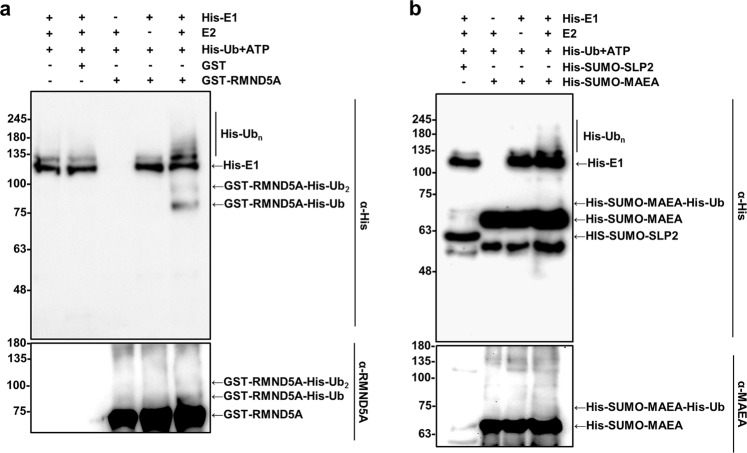
Recombinant RMND5A and MAEA exhibit E3 ligase activity *in vitro*. (**a**) Bacterially expressed RMND5A exhibits E3 ligase activity. Bacterial extracts with induced expression of GST (lane 2) or GST-RMND5A (lane 6) were incubated with ATP and His-ubiquitin and with or without, as indicated, His-E1 and UBE2D2 (E2) for 2 hours at 37 °C. Reactions were analyzed by Western blot using anti-His antibody (top), or an RMND5A antibody (bottom). The identity of the products is indicated on the right. (**b**) SUMO-MAEA has *in vitro* ligase activity. His-tagged SUMO MAEA was expressed and incubated in reaction as in. (**a**) His-tagged SUMO-SLP2 was used as a negative control. Analysis was done by Western blot analyzed using an anti-His antibody (top), or a MAEA antibody (bottom).

To further characterize the E3 ligase activity of the RING domain subunits RMND5A and MAEA, we conducted *in vitro* ubiquitination assays with bacterially expressed proteins. Initial experiments using purified GST-RMND5A and SUMO-MAEA failed to show any detectable activity (data not shown), possibly due to poor folding or insolubility of these enzymes. Therefore, we omitted the purification step and conducted E3 ligase assays using crude bacterial extracts as previously done to test the activity of Gid2 and Gid9^7,11^. In these conditions, human GST-RMND5A exhibited weak, but observable substrate and self-ubiquitination activity (Fig. 5a).

Previous studies reported that the bacterially expressed yeast Gid9, the homologue of MAEA, had no detectable E3 ligase activity *in vitro*^11^. However, *in vivo*, a cysteine mutation in the MAEA RING domain abolished Gid complex ubiquitination of FBPase, suggesting that the RING domain of Gid9 is required for the Gid complex activity^11^. Surprisingly, a SUMO tagged recombinant version of human MAEA expressed in *Escherichia coli* exhibited some E3 ubiquitin ligase activity (Fig. 5b), albeit weaker than that of RMND5A. Thus, both recombinant RMND5A and MAEA display E3 ligase activity *in vitro*.

### Characterization of E2 pairings and lysine linkage

**Figure 6:**
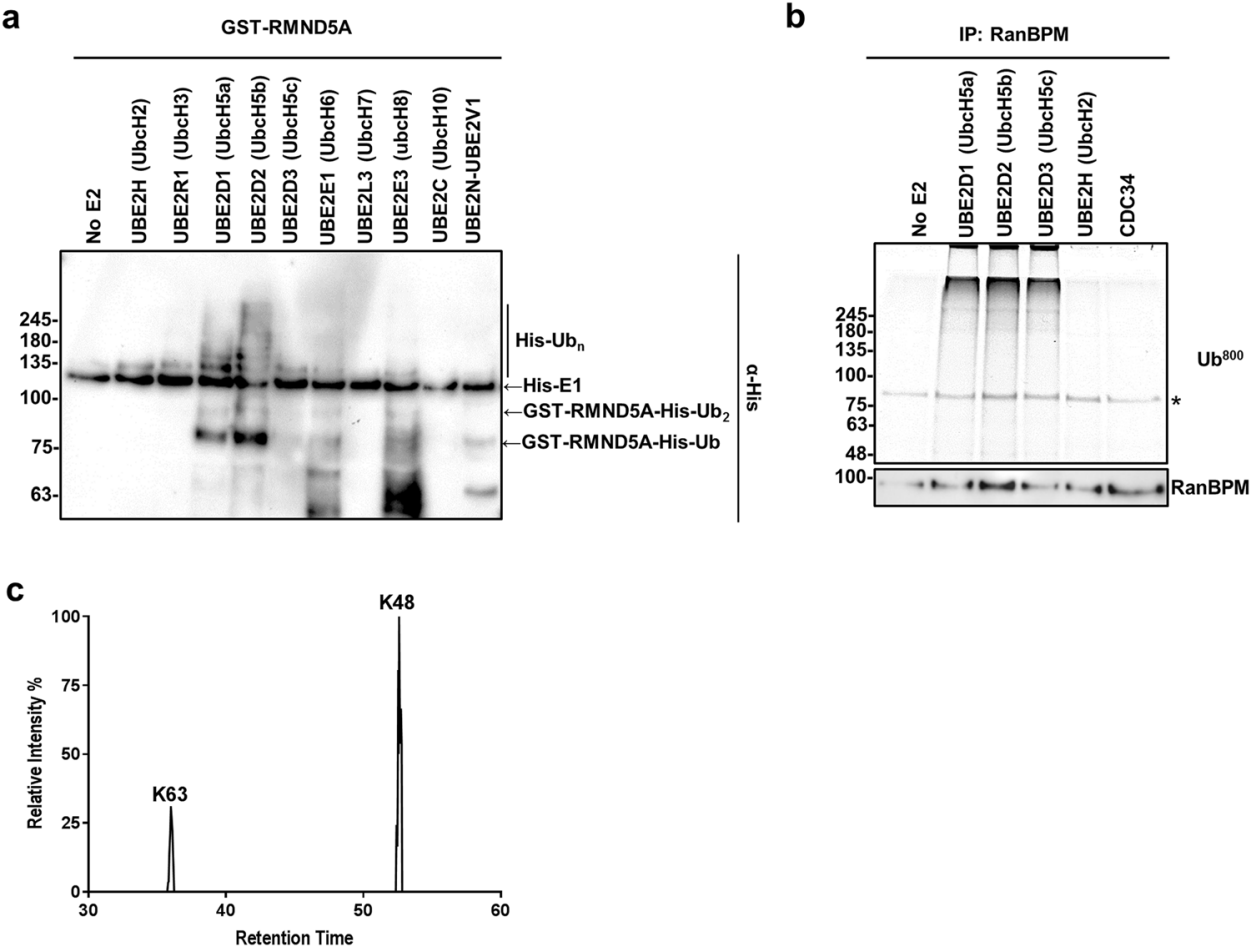
Properties of the CTLH complex E3 ligase activity. (**a**) E2 enzymes that function with GST-RMND5A. GST-RMND5A ubiquitination assays were conducted as described in Fig. 5a with the indicated E2 enzymes. Uncropped image and GST empty vector reactions are shown in Supplementary Fig. 5. (**b**) RanBPM immunocomplexes functionally couple with UBE2D1, UBE2D2 and UBE2D3 but not with UBE2H or CDC34. RanBPM was immunoprecipitated from HEK293 cell extracts, resuspended in ubiquitin assay master mix containing flourescein-ubiquitin and the indicated E2 enzymes were added and incubated at 37 °C for 30 minutes. Reactions were run on SDS-PAGE gel and fluorescein-ubiquitin was imaged directly on the gel using a LICOR imager at 800 nm. Asterisk indicates a non-specific band. (**c**) GST-RMND5A mediates K48 and K63 polyubiquitination. GST-RMND5A ubiquitination assay reactions (as in Fig. 5a) were LysC/trypsin digested and then subjected to analysis by liquid chromatography tandem Mass Spectrometry (LC-MS/MS). Shown is a representative replicate of the MS1 peak extraction for ubiquitin peptides containing GlyGly sites. See also Supplementary Fig. 6.

As E3 ligase ubiquitination activity is dependent on a specific E2 enzyme, we sought to determine which E2 enzymes function optimally with the CTLH complex. In a panel of 11 E2 enzymes, the GST-RMND5A fusion protein exhibited E3 ligase activity only when UBE2D1 (UbcH5a) or UBE2D2 (UbcH5b) are present in the reaction (Fig. 6a, Supplementary Fig. 5), while RanBPM immunocomplexes were able to function with UBE2D1, UBE2D2 and UBE2D3 (Fig. 6b). Interestingly, the complex or GST-RMND5A did not exhibit activity when paired with UBE2H (UbcH2) (Fig. 6a,b), the human homologue of yeast Gid3^28^ (also known as ubc8), which is the E2 required for the glucose-induced ubiquitination of FBPase^29,30^. Similarly, no activity was detected with CDC34 (Fig. 6b), which has a C-terminal extension of acidic residues similar to that of Gid3 that is critical for its activity^31^.

To assess which type of chain linkage is being mediated by RMND5A when paired with UBE2D2, we trypsin/LysC digested the GST-RMND5A ubiquitination assay reactions and analyzed the modified ubiquitin peptides using mass spectrometry. In the GST-RMND5A assays with UBE2D2, approximately 80% of modified ubiquitin was ubiquitinated on K48, while the remaining 20% was K63 (Fig. 6c, Supplementary Fig. 6). No other lysine linkages were detected. This suggests that RMND5A can mediate both K48 and K63 ubiquitin chains, with a preference for K48.

### Muskelin is a target of the CTLH complex

**Figure 7:**
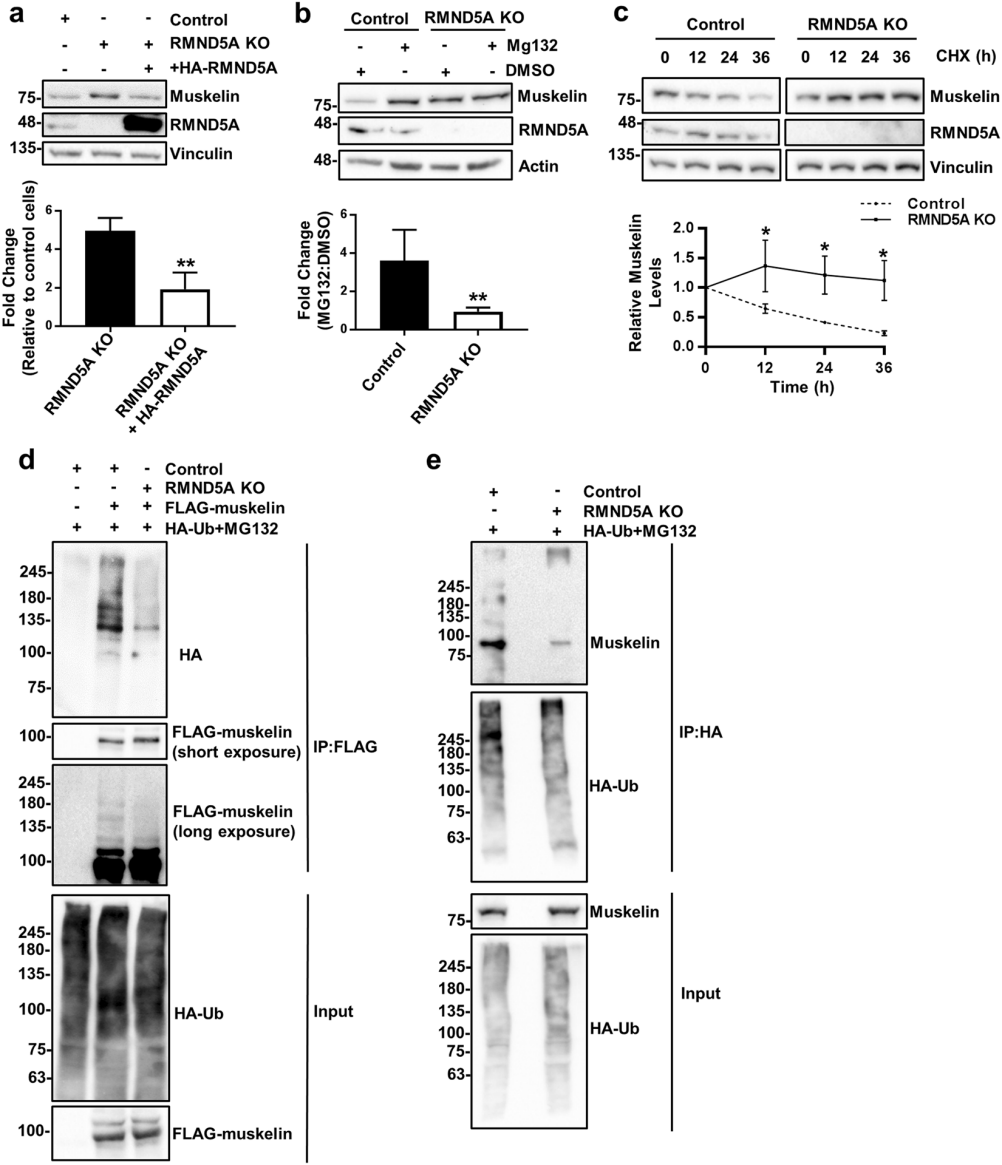
Muskelin is a target of the CTLH complex. (**a**) Deregulation of muskelin in RMND5A KO cells can be rescued by restoration of RMND5A expression. HeLa whole cell extracts prepared from control, RMND5A KO, or RMND5A KO cells transfected with HA-RMND5A for 24 hours were analyzed by Western blot with the antibodies indicated. *Below*, quantification of muskelin levels is shown to the right for untransfected (−) or HA-RMND5A transfected RMND5A KO HeLa cell, normalized to vinculin levels and relative to control cells set to 1. N = 3, error bar indicates SD. **P < 0.01. (**b**) Muskelin upregulation is proteasome-dependent. Whole cell extracts were prepared from Control or RMND5A KO HeLa cells treated with DMSO or 10 μM MG132 for 16 hours and analyzed by Western blot. *Below*, quantifications of muskelin in MG132-treated control or RMND5A KO HeLa cells, normalized to actin levels and relative to their DMSO control set to 1. N = 4, error bar indicates SD. **P < 0.01. (**c**) Degradation kinetics of muskelin. Control or RMND5A KO HeLa cells were treated with 100 μM cycloheximide (CHX) and whole cell extracts were prepared at the times indicated. Samples were run on a 10% SDS-PAGE gel and analyzed by Western blot with the indicated antibodies. Muskelin quantifications are shown below relative to vinculin levels and normalized to untreated samples which were set to 1. N = 3, error bars indicate SD. *P < 0.05. The error bar for the KO samples at 24 h is not visible because it is smaller than symbol size. (**d**) Ubiquitination of transfected muskelin is regulated by RMND5A. Control or RMND5A KO HEK293 were co-transfected with FLAG-muskelin or HA-ubiquitin (HA-Ub) for 24 hours, followed by 10 μM MG132 treatment for 8 hours. FLAG-muskelin was pulled down in denaturing conditions. Note that transfected muskelin migrates higher than endogenous muskelin due to the added tags that amount to about 7 kDa. (**e**) Ubiquitination of endogenous muskelin is regulated by RMND5A. Control or RMND5A KO HEK293 were transfected with HA-Ub for 24 hours, followed by 10 μM MG132 treatment for 8 hours. HA-Ub was pulled down in denaturing conditions.

Muskelin is the only member of the CTLH complex that does not have a yeast homologue in the Gid complex and therefore stands out as a unique difference between the two complexes^10^. Interestingly, muskelin protein levels were significantly increased in both RMND5A and MAEA KO cells compared to control HeLa cells and this was reversed by the reintroduction of RMND5A and MAEA into their respective knockout cells by transient transfection (Figs 3c,d and 7a and Supplementary Fig. 3). Treatment with the proteasome inhibitor MG132 resulted in increased muskelin levels in control cells relative to DMSO control treatment, but not in RMND5A KO HeLa cells (Fig. 7b), suggesting that the increase in muskelin in RMND5A KO cells is proteasome dependent. To confirm that muskelin protein stability was dependent on the CTLH complex, we performed cycloheximide (CHX) treatments to compare muskelin degradation of WT and RMND5A KO cells. Muskelin had a half-life of about 24 hours in WT cells, whereas no significant degradation was detected in RMND5A KO cells, even 36 hours following addition of CHX, suggesting that muskelin degradation is dependent on the CTLH complex activity (Fig. 7c). This led us to suspect that muskelin could be a ubiquitination target of the complex leading to proteasomal degradation.

To evaluate if ubiquitination of muskelin is regulated by the CTLH complex, we co-transfected HA-ubiquitin and a construct encoding FLAG-muskelin in control or RMND5A KO HEK293 cells, followed by treatment with MG132. FLAG pulldown of transfected muskelin and hybridization with an HA antibody showed a poly-ubiquitination pattern in control cells which was reduced to background levels in RMND5A KO cells (Fig. 7d). Furthermore, endogenous ubiquitinated muskelin co-immunoprecipitated with transfected HA-ubiquitin in control cells but not in RMND5A KO cells (Fig. 7e). Together, this suggests that the CTLH complex is required for muskelin ubiquitination *in vivo*. Overall, the data suggests that the CTLH complex regulates ubiquitination and protein levels of one of its own subunits, muskelin.

## Discussion

In this study, we have characterized the composition and stability of the CTLH complex and demonstrated, using *in vitro* assays, that the complex has E3 ligase activity. We confirmed that human GID4 and WDR26 associate with the complex and that RanBPM, TWA1, MAEA, RMND5A and ARMC8 each have roles in maintaining complex stability. Importantly, RMND5A and MAEA are completely co-dependent on each other. We found that ubiquitination activity of the complex is dependent on the RING subunits RMND5A and MAEA. Furthermore, both recombinant RMND5A and MAEA also exhibit activity. Additionally, we determined that the complex can pair with ubcH5 family of E2 enzymes and GST-RMND5A can catalyze K48 and K63 ubiquitin chains. Finally, we revealed that CTLH complex regulates ubiquitination and proteasomal degradation of its peripheral subunit muskelin.

Our data suggests that protein expression of several CTLH complex subunits is interdependent, which has previously been observed to some extent for the yeast Gid complex. In the Gid complex, Gid1 (RanBPM) was deemed essential for the stability of the complex as its deletion resulted in decreased levels of Gid8 and Gid2^26^. Also, Gid2 and Gid9 were found to stabilize each other^26^. Our analyses of CTLH complex subunits knockdown/knockout cells suggest that this is also true for the CTLH complex. RanBPM knockdown resulted in the decrease of TWA1, RMND5A and MAEA protein levels and RMND5A and MAEA required each other, suggesting that, like in the Gid complex, the two RING domain subunits are stabilized through heterodimerization. TWA1, like RanBPM, is central to CTLH complex formation, as its knockout affected RanBPM, MAEA, RMND5A and, curiously, ARMC8β. Finally, muskelin knockout did not significantly affect other CTLH complex members’ protein levels. Previous microarray analyses revealed no changes in CTLH complex members’ RNA expression in RanBPM shRNA cells^32^, and this was confirmed through qPCR analyses. We did not detect any significant changes in mRNA levels in most other CTLH complex knockout cells, except in 2 cases (RMND5A and ARMC8) where slight changes in mRNA expression were detected but were much more subtle than the effect observed at the protein level. Therefore the changes in protein expression induced by the knockout of individual CTLH subunits reflect mostly changes in protein stability rather than at the transcriptional level.

We found that both ARMC8α and β were present in the CTLH complex. These 2 isoforms originate from alternative splicing of the same gene product and have previously been identified as being part of the CTLH complex^13^. However, it raises the question as to whether both isoforms are present together in the complex or that sub-complexes contain one or the other isoform. Interestingly, the loss of TWA1 had different effects on the two ARMC8 isoforms, with ARMC8β being strongly decreased and ARMC8α slightly increased, albeit not significantly, inferring that the alternate splicing event may be regulated by the CTLH complex. Reversely, ARMC8 knockout significantly reduced TWA1, MAEA and RMND5A levels, suggesting that ARMC8 may stabilize the association of the core complex members.

Immunofluorescence analyses showed that the CTLH complex subunits are localized to different extents in the cytoplasm and nucleus. The subcellular localization observed with ectopically expressed proteins appear to match that of several endogenously expressed CTLH complex members reported in a previous study^13^. Adding to that, we found that GID4 is nearly exclusively nuclear, whereas WDR26 is mostly cytoplasmic. This suggests the possibility that several distinct CTLH complex variants exist in the cell. However, the fact that the knockout of MAEA, which is mostly nuclear, affects the levels of muskelin, which is mostly cytoplasmic, implies that these two seemingly differentially localized members are connected. Therefore, the dynamics of the nucleocytoplasmic localization of CTLH complex(es) will need further investigation. Also, our analysis involved ectopically expressed proteins, and while our results show that transfected CTLH complex members can interact with the endogenous CTLH complex (see Fig. 1), it is possible that a significant fraction of the transfected proteins did not associate with the complex and that their observed localization was dictated by their own localization properties rather than by those of the complex as a whole.

Our analyses of the CTLH complex E3 activity through *in vitro* ubiquitination assays using RanBPM immunocomplexes showed that, as expected, RMND5A is essential for activity. MAEA is also likely essential since its KO reduces RMND5A protein levels to background, therefore preventing CTLH complex activity. Also, based on the yeast topology, MAEA links TWA1 to RMND5A and potentially ARMC8 and therefore is essential for complex formation^26^.

We found that both bacterially-expressed recombinant RMND5A and MAEA have E3 ligase activity. This was previously reported for the *Xenopus laevis* and yeast homologues of RMND5A^7,12^, however, this is the first time that MAEA is shown to have intrinsic activity. The activity of both RMND5A and MAEA was low, and the reason for this may be that both contain RING domains that diverge considerably from the RING consensus^10^. Analysis of the consensus sequence for RMND5A homologues suggests its RING domain resembles that of TRIM32, but contains serines instead of cysteines in its second active site^10^. TRIM32 exists as a tetramer formed by two separate dimerization events that occur through the RING domains and the coiled‐coil region^33^. TRIM32 RING dimerization is essential for catalytic activity^33,34^. This suggests that this may also be true for the RMND5A-MAEA dimer. Previous analyses with the yeast complex found that the interaction of Gid2 and Gid9 still occurs when the Gid2 CTLH and LisH domains are deleted, suggesting that the interaction may be mediated by the RING domains^11^. In our *in vitro* assays, we expressed RMND5A as a fusion protein with GST. Since GST dimerizes, that could be fulfilling the pre-requisite for dimerization. Alternatively, the LisH and CTLH domains could mediate dimerization and/or the RING domain may have similar dimerization capabilities as TRIM32. The MAEA RING domain is longer than conventional RING domains, is not predicted to coordinate zinc and has no obvious similarities to other eukaryotic RING motifs^10^. As it was expressed as a SUMO-fusion protein, dimerization, if required for activity, could only have been mediated by the RING and/or the LisH/CTLH domains.

Another study recently described the activity of the CTLH complex through biochemical reconstitution with purified components^35^. While some of our conclusions are in agreement with their findings, notably with respect to the requirements for MAEA and the other core complex subunits RanBPM, TWA1 and RMND5A for E3 ligase activity, some of our results differ, in particular regarding the identification of the E2 requirement for complex activity. While we detected robust activity with UBE2D1, UBE2D2 and UBE2D3 variants, we did not observe any activity with UBE2H, or CDC34. In agreement with our data, Lampert *et al*. did not report any activity with CDC34, however, they found that UBE2H was the most efficient E2 and they observed little activity with UBE2D3. While UBE2H was identified as the human homologue of Gid3^28^, it lacks the C-terminal extension of acidic residues found in Gid3. This acidic tail is reminiscent of CDC34 in which the tail both binds the E3 ligase and promotes ubiquitin transfer^31^. Most of their assays involved the *in vitro* reconstituted CTLH complex with subunits purified from insect cells, whereas our assays involved bacterially-expressed RMND5A and the endogenous CTLH complex immunoprecipitated from HEK293 cells. Another difference is that we assayed complex activity from endogenous RanBPM immunoprecipitates, whereas they assayed the E3 activity from the complex immunoprecipitated through transfected tagged-RMND5A. The use of different experimental models (recombinant versus native complex) or the difference in complex isolation procedure (potentially yielding different complex conformations) as well as the relative activities of E2 enzymes could potentially account for these differences. However, our *in vitro* assays using recombinant RMND5A are consistent with previous *in vitro* ubiquitination assays which showed that yeast Gid2 (homolog of RMND5A) was able to function with UBE2D2^7^.

Our finding that muskelin protein levels and ubiquitination are regulated by the CTLH complex provides insight into a possible function and/or regulatory module of the complex. It also raises the question as to whether muskelin has a role in CTLH complex function or if it is just one of its targets. If the former, its appearance later in evolution suggests that it may provide a function to the complex distinct from the yeast Gid complex. As shown in this study and others, muskelin localizes mostly to the cytosol, which is dependent on its LisH domain-mediated dimerization^13,20,36–38^. Interestingly, its overexpression has previously been shown to promote relocalization of other CTLH nuclear complex members to the cytoplasm^13^. Therefore, we speculate that the function of muskelin in the CTLH complex may be to direct the complex to specific cytoplasmic targets. Ubiquitination and subsequent degradation of muskelin by the complex could therefore serve as a feedback mechanism under certain conditions. Self-ubiquitination is a common feature of most E3 ligases and in many cases is used as a mechanism of autoregulation^39,40^.

Muskelin promotes cellular prion protein (PrP^C^) turnover via the lysosome in neurons and muskelin knockout accelerates Prion disease induced by prion infection in mice^19^. Interestingly, a fragment of RanBPM is found overexpressed in Alzheimer’s Disease (AD) patients and its overexpression promotes Amyloid beta (Aβ) generation and hallmarks of AD^41–45^. As PrP^C^ propagates the neurotoxic signaling effects of Aβ^46–48^, this could implicate a mechanism whereby the effects observed by RanBPM overexpression in AD are, at least partially, a result of increased ubiquitination and proteasomal degradation of muskelin, leading to a defect in PrP^C^ lysosomal degradation. It will be of interest to assess adult mouse models of other CTLH complex members, especially in the context of neurodegenerative diseases, to determine if there is an interplay between the CTLH complex and muskelin ubiquitination that contributes to disease pathogenesis.

In summary, we have extended the characterization of the mammalian CTLH complex, a unique E3 ligase complex containing a RING heterodimer. We confirmed its function as an E3 ubiquitin ligase and determined the requirements for its stability and activity. We have also revealed it regulates ubiquitination of its subunit muskelin, adding to its list of possible targets and identifying a potential autoregulatory mechanism that awaits further investigation. A comprehensive study of the substrates of the CTLH complex will be informative to understand the biological function of this multi-subunit E3 ubiquitin ligase.

## Methods

### Cell culture, transfections and treatments

Wild-type HeLa and HEK293 cells, and control shRNA and RanBPM shRNA stable HEK293 cells have been described previously^49,50^. All cells were cultured in high glucose Dulbecco’s modified Eagle’s medium (Wisent Bioproducts, St. Bruno, Quebec, Canada) supplemented with 10% fetal bovine serum (Wisent Bioproducts) at 37 °C in 5% CO_2_. Cells were treated with 10 μM MG132 (EMD-CalBiochem, San Diego, CA) for the indicated time points. For cycloheximide (CHX) treatment, cells were treated with 100 μg/ml cycloheximide (BioShop, Burlington, ON, Canada) and collected at the indicated time points. Plasmid transfections were carried out with jetPRIME (Polypus Transfection, Illkirch, France) according to the manufacturer’s protocol. siRNA transfections were carried out as described previously^51^ for siMuskelin and siRMND5A and siTWA1 (Silencer, AM16708A, 25822, Ambion, Life Technologies, Burlington, ON, Canada) transfections were performed using the same conditions.

### Generation of CRISPR Knockout cells

Single guide RNA (sgRNA) sequences were designed using the Benchling CRISPR tool (https://benchling.com). Top and bottom oligos with overhanging ends containing sgRNA directed against RMND5A (5′CACCGTGGAGCACTTCTTTCGACA, 5′AAACTGTCGAAAGAAGTGCTCCAC) MAEA (5′CACCGCGTTTGTTCAGCGTCTCGTA), TWA1 (5′CACCGAGCAGCGGAGAAGTTTCGAA, 5′AAACTTCGAAACTTCTCCGCTGCTC), ARMC8 (5′CACCGTTTGGTTCGAATGTGCAGTA, 5′AAACTACTGCACATTCGAACCAAAC), RanBPM (5′CACCGCGAAGGCTTAGGGGCCGCGG, 5′AAACCCGCGGCCCCTAAGCCTTCGC), or muskelin (5′CACCGCTATTTTAATGAATCGCACA, 5′AAACTTGTCGATTCATTAAAATAGC) were cloned into pSpCas9(BB)-2A-Puro V2.0 (PX459) digested with BpiI and then transfected into early passage HEK293 or HeLa cells. Forty-eight hours after transfection, cells were put under puromycin selection (1.2 μg/ml for HEK293, 0.3 μg/mL for HeLa) for seven days, followed by colony picking and expansion. Control cells were generated by the above approach but using Cas9 alone (i.e. without an sgRNA guide) and pooling colonies together.

### Plasmid constructs

The following plasmids were gifts: pCDNA-SBP-FLAG-WDR26^51^ (Dr. Songhai Chen, University of Iowa, Iowa City, IA, USA), pMT123 plasmid expressing HA-ubiquitin^52^ (Dr. Lina Dagnino, Western University, London, ON, Canada), pET-His-SUMO-SLP2 (Dr. Stan Dunn, Western University, London, ON, Canada), pSpCas9(BB)-2A-Puro V2.0 (PX459; Dr. Joe Torchia, Western University, London, ON, Canada) and pCMV-HA-muskelin^53^ (mouse cDNA; Dr. Kai Jiao, University of Alabama at Birmingham, Birmingham, AL, USA). pCMV-HA-RanBPM has been described previously^49^. Primers for RMND5A (5′GTACATTCTAGAATGGATCAGTGCGTGACGGTGGAG, 5′GTACATGGATCCTCAGAAAAATATCTGTTTGGCATC), MAEA (5′GTACATTCTAGAATGGCGGTGCAGGAGTCGGCGGCTCAGTTGTCC, 5′GTACATGGATCCCTACATGATGTACACCTTCTCGGCTTGTGAGAAG), TWA1 (5′GTACATTCTAGAATGAGTTATGCAGAAAAACCCGATG, 5′GTACATGGATCCCTACTTGGGCTCCTCAATCACACCC), were used to generate inserts amplified from a Jurkat T-cell cDNA library (Clontech, see reference^54^). The inserts were digested with XbaI and BamHI enzymes and cloned into pCGN-HA plasmid (created by removal of Oct-1 from pCGN-Oct-1, see reference^55^). ARMC8 insert was generated by PCR amplification (5′ATTGAAGTCGACGATGGAAGTAACAGCTAGCAGTCG, 5′AATGTTCTCGAGATTGCCAGGTACTGCTGCAGTGC) from the same cDNA library, digested with SalI and XhoI and ligated with pCMV-HA (created by removal of RanBPM from pCMV-HA-RanBPM). Muskelin cDNA (derived from pCMV-HA-muskelin (mouse)) was PCR amplified (5′ATTATAACCGGTATGGCGGCTGGCGGGGCTGTTG, 5′AGTCCGCTCGAGCTACAGTGTGATCAGGTCTACCAG) with Age1 and XhoI sites and subcloned into pCDNA-SBP-FLAG (created by removal of WDR26 from pCDNA-FLAG-SBP-WDR26). GID4 cDNA (Accession: NM_024052) in pEZ-M06 was purchased from GeneCopoeia (EX-E1982-M06; Rockville, MD, USA). RMND5A cDNA was PCR amplified from pCGN-HA-RMND5A (5′GTACATGAATTCATGGATCAGTGCGTGACGGTGGAG, 5′GTACATGGATCCTCAGAAAAATATCTGTTTGGCATC), digested with EcoRI and BamHI and subcloned into pGEX-4T1. MAEA cDNA was PCR amplified from pCGN-HA-MAEA (5′TATTTAGGTACCATGGCGGTGCAGGAG, 5′GCCGTAAAGCTTCTACATGATGTACACCTTCTCGGC), digested with KpnI and HINDIII and subcloned into pET-His-SUMO (created by removal of SLP2 from pET-His-SUMO-SLP2).

### RNA extraction, reverse transcription and quantitative PCR

RNA extraction was performed using RNeasy Mini Kit (QIAGEN, Toronto, Canada) in accordance with the manufacturer’s directions. 2 μg of total RNA was reverse transcribed to cDNA using the High Capacity cDNA Reverse Transcription Kit (Applied Biosystems, Foster City, California, United States). Quantitative PCR was completed on Bio-Rad CFX Connect Real-Time System (Bio-Rad) using pre-designed TaqMan probes (4448892, Applied Biosystems) for the following genes: RANBP9 (Hs00170646_m1), GID8 (Hs00215479_m1), RMND5A (Hs00405598_m1), MKLN1 (Hs00992683_m1), MAEA (Hs01028524_m1), and ARMC8 (Hs01046446_m1). Experiments were performed with technical triplicates using GAPDH (4453320, Hs02786624_g1, Applied Biosystems) as a normalization control. Changes in gene expression were determined by the Δ(ΔCt) method and reported as fold increase or decrease in mRNA levels in knockout cell line samples relative to control cells.

### Western blot

Whole cell extracts were prepared as described^49^ and resolved using 8% or 10% SDS-polyacrylamide gel electrophoresis (SDS-PAGE). Gels were transferred to polyvinylidene fluoride membranes, blocked in 5% milk and hybridized with the following antibodies: ARMC8 (E-1, sc-365307; Santa Cruz Biotechnology, Santa Cruz, CA, USA); FLAG (M2, F1804, Sigma-Aldrich, St. Louis, MO, USA); HA (HA-7, H3663 Sigma-Aldrich); MAEA (AF7288, R&D Systems, Minneapolis, MN, USA); Muskelin (C-12, sc-398956, Santa Cruz Biotechnology); RanBPM (5M, 71-001, Bioacademia, Japan); RMND5A (NBP1-92337, Novus Biologicals, Littleton, CO, USA); TWA1 (NBP1-32596, Novus Biologicals); Vinculin (E1E9V, Cell Signaling Technology, Danvers, MA, USA); WDR26 (ab85962, Abcam, Cambridge, UK); β-actin (A5441, Sigma-Aldrich) and His (MAB050, R&D Systems). Blots were developed using Clarity Western ECL Substrate (BioRad, Hercules, CA, USA) and imaged using a ChemiDoc MP (BioRad). Analysis and quantifications were done using Image Lab (BioRad).

### Immunofluorescence

Cells were plated on coverslips and transfected following overnight incubation. Cells were fixed with 3% paraformaldehyde, permeabilized in 0.5% Triton-X100 for 10 min and blocked in 5% FBS diluted in PBS. Coverslips were incubated overnight with primary antibody HA (HA-7, H9658, Sigma-Aldrich) or FLAG (M2, F1804, Sigma-Aldrich), washed in PBS and incubated with anti-mouse Alexa Fluor 488 secondary antibody (Invitrogen, Life Technologies, Burlington, ON, Canada). Cells were mounted with ProLong Gold antifade with DAPI (Invitrogen). Visualization was done using an Olympus BX51 microscope with a 40x objective and images were captured with the Image-Pro Plus software (Media Cybernetics Inc., Bethesda, MD, USA). For quantification analysis, images were blinded by a third party.

### Immunoprecipitations

For co-immunoprecipitation experiments, extracts were adjusted to 0.25% NP-40, pre-cleared and rotated overnight at 4 °C with antibodies to RanBPM (F-1, sc-271727, Santa Cruz Biotechnology), HA (HA-7, H9658, Sigma-Aldrich), or FLAG (M2, F1804, Sigma-Aldrich). Samples were then incubated for 1 hour at 4 °C with Dynabeads protein G (10004D, Invitrogen), washed three times in lysis buffer and resuspended in SDS loading buffer. Mouse IgG (sc-2025, Santa Cruz Biotechnology) was used as negative control for RanBPM immunoprecipitations. For ubiquitination assays, RanBPM immunoprecipitations were carried out as above, except extracts were supplemented with extra NP-40 (final 1.0%) and Triton (final 0.2%) and washed three times in lysis buffer without EDTA.

To assess ubiquitination of FLAG-muskelin, control or RMND5A KO HEK293 cells were co-transfected with pCDNA-FLAG-muskelin and pMT123 plasmid expressing HA-ubiquitin^52^. Twenty-four hours after transfection, cells were MG132 treated for 8 hours. Cells were lysed in denaturing buffer (50 mM Tris, pH 7.5, 150 mM NaCl, 1% Triton, 1% SDS, 1 mM Na3VO4, 10 mM NaF, 1 mM phenylmethylsulfonyl fluoride (PMSF), 1 μg/ml of aprotinin, 10 μg/ml of peptatin, 1 μg/ml of leupeptin and 25 mM NEM (N-Ethylamalide, Bioshop Canada, Burlington, ON, Canada)), passed through a 23 G needle ten times and incubated on ice for 30 minutes. For immunoprecipitation, lysates were diluted 1:10 in buffer A (50 mM Tris, pH 7.5, 150 mM NaCl, 1% Triton, 1 mM Na3VO4, 10 mM NaF and 25 mM NEM) and incubated with anti-FLAG (M2; F1804; Sigma-Aldrich) for 2 hours at 4 °C, followed by incubation with Dynabeads Protein G for 1 hour. Beads were then washed five times in buffer A and resuspended and boiled in SDS loading dye. Eluates were split in half when loading on SDS-PAGE so that HA and FLAG immunoblotting could be analyzed separately (to avoid overlapping signal). The same procedure was employed for the HA-ubiquitin immunoprecipitation, except that cells were transfected with only HA-ubiquitin plasmid and anti-HA (HA-7, H9658, Sigma-Aldrich) was used for the pulldown.

### *In vitro* ubiquitination assays

BL21 *E. coli* expressing GST, GST-RMND5A, His-SUMO-SLP2 and His-SUMO-MAEA were grown at 37 °C with 250 μM ZnCl_2_ to 0.7–0.8 OD600 and induced with 0.5 mM Isopropyl β-D-1-thiogalactopyranoside, then grown at 16 °C overnight. Cells were lysed in 50 mM Tris–HCl, pH 7.5, 250 mM NaCl, 5 mM dithiothreitol (DTT), 2 mM PMSF, 20 μg/mL Leupeptin, 1.0 μg/mL aprotinin and 1% Triton-X 100 and passed through a French press twice. 4.5 μL of bacterial extract was used in a 20 μL ubiquitination reaction with 5 μM UBE2D2 (E2-622, BostonBiochem, Cambridge, MA), 0.1 μM His-tagged E1^56^, 25 μM His-ubiquitin, 10 mM Mg-ATP (B-20, R&D Systems), 50 mM Tris-HCl, pH 7.5, 50 mM NaCl, 10 mM MgCl2, 0.1 mM DTT and 25 μM ZnCl_2_. The UbcH (E2) Enzyme Kit (K-980B, BostonBiochem) and CDC34 (see reference^57^) was used to screen E2 enzymes. Reactions were incubated in a thermomixer with periodic shaking at 37 °C for 2 hours. The entire reactions were run on an 8% SDS-PAGE and analyzed by Western blot. For ubiquitination assays with RanBPM immunocomplexes, beads were resuspended in 0.1 μM His-tagged E1, 5 μM UbcH5b, 22.5 μM His-ubiquitin, 2.5 μM Ub^800^ (see reference^58^), 10 mM Mg-ATP and 50 mM HEPES. Reactions were incubated in a thermomixer with periodic shaking at 37 °C for 30 minutes. Gels were scanned by the Odyssey Imaging system (LiCor, Lincoln, NE, USA) and fluorescence intensity was measured at 800 nm.

### Chain linkage analysis by Mass Spectrometry (MS)

Sample preparation of GST-RMND5A ubiquitination reactions were performed as described previously^59,60^. Briefly, reactions were reduced with 10 mM DTT, alkylated with 100 mM Iodoacetamide, followed by methanol precipitation. The protein pellet was resuspended in 0.5% PPS Silent Surfactant (Expedeon Ltd, Cambridge, UK) and digested for 16 hours with 0.5 mg of Lysyl Endopeptidase (LysC; 125-05061, Wako Pure Chemical Ind., Ltd., Japan) and 0.75 mg of LysC/Trypsin (V5071, Promega, Madison, WI, USA), followed by 4 hours with 0.5 mg Trypsin (V5111, Promega). Quenched digested reactions were then desalted with C18 Stage Tips and eluted peptides were lyophilized, followed by resuspension in 0.1% Formic acid. Samples (1 μg as measured by BCA) were analyzed by using an M-class nanoAquity UHPLC system (Waters, Milford, MA, USA) connected to a Q Exactive mass spectrometer (Thermo Scientific, Waltham, MA, USA). Raw MS files were searched in MaxQuant (version 1.5.8.3) using the Human Uniprot database. MS1 peak areas corresponding to each ubiquitin linkage were extracted using Xcalibur software (Thermo Scientific).

### Statistical analysis

Statistical analyses were performed using GraphPad PRISM (GraphPad Software Inc., La Jolla, CA, USA). Differences between two groups were compared using unpaired two-tailed t test. Results were considered significant when *P* < 0.05.

## Supplementary information

Supplementary Figures 1 to 6 and Uncropped Blots
