# Supplementary material for: The mammalian CTLH complex is an E3 ubiquitin ligase that targets its subunit muskelin for degradation
Source: Sci Rep. 2019 Jul 8;9:9864. doi: 10.1038/s41598-019-46279-5 (PMC6614414; doi:10.1038/s41598-019-46279-5)

SUPPLEMENTARY FIGURES
accompanying the manuscript entitled

The mammalian CTLH complex is an E3 ubiquitin ligase that targets its subunit muskelin for degradation

Matthew E.R. Maitland[1, 2, 3], Gabriel Onea[1, 2], Christopher A. Chiasson[1, 2], Xu Wang[1], Jun Ma[2,3], Sarah E. Moor[1, 2], Kathryn R. Barber[2], Gilles A. Lajoie[2, 3], Gary S. Shaw[2], and Caroline Schild-Poulter[1, 2, *]

[1]Robarts Research Institute, Schulich School of Medicine & Dentistry, The University of Western Ontario, London, Ontario, Canada.

[2]Department of Biochemistry, Schulich School of Medicine & Dentistry, The University of Western Ontario, London, Ontario, Canada.

[3]Don Rix Protein Identification Facility, Schulich School of Medicine & Dentistry, The University of Western Ontario, London, Ontario, Canada

[*]Correspondence: cschild-poulter@robarts.ca.

Supplementary Figure 1. MAEA and Muskelin co-immunoprecipitate with RanBPM in stringent binding conditions. HEK293 whole cell extracts were adjusted to the indicated final detergent concentrations and then incubated with a RanBPM antibody and immunoprecipitated. Immunoprecipitates were analyzed by Western blot with the indicated antibodies. IgG was used as a negative control.

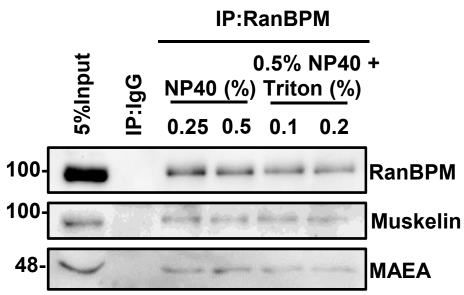

Supplementary Figure 2. Analysis of mRNA levels of proteins that change in figure 3. Fold changes shown are relative to control cells, and normalized to GAPDH levels. Details described under “Methods”. n=3, *p<0.05, **p<0.01.

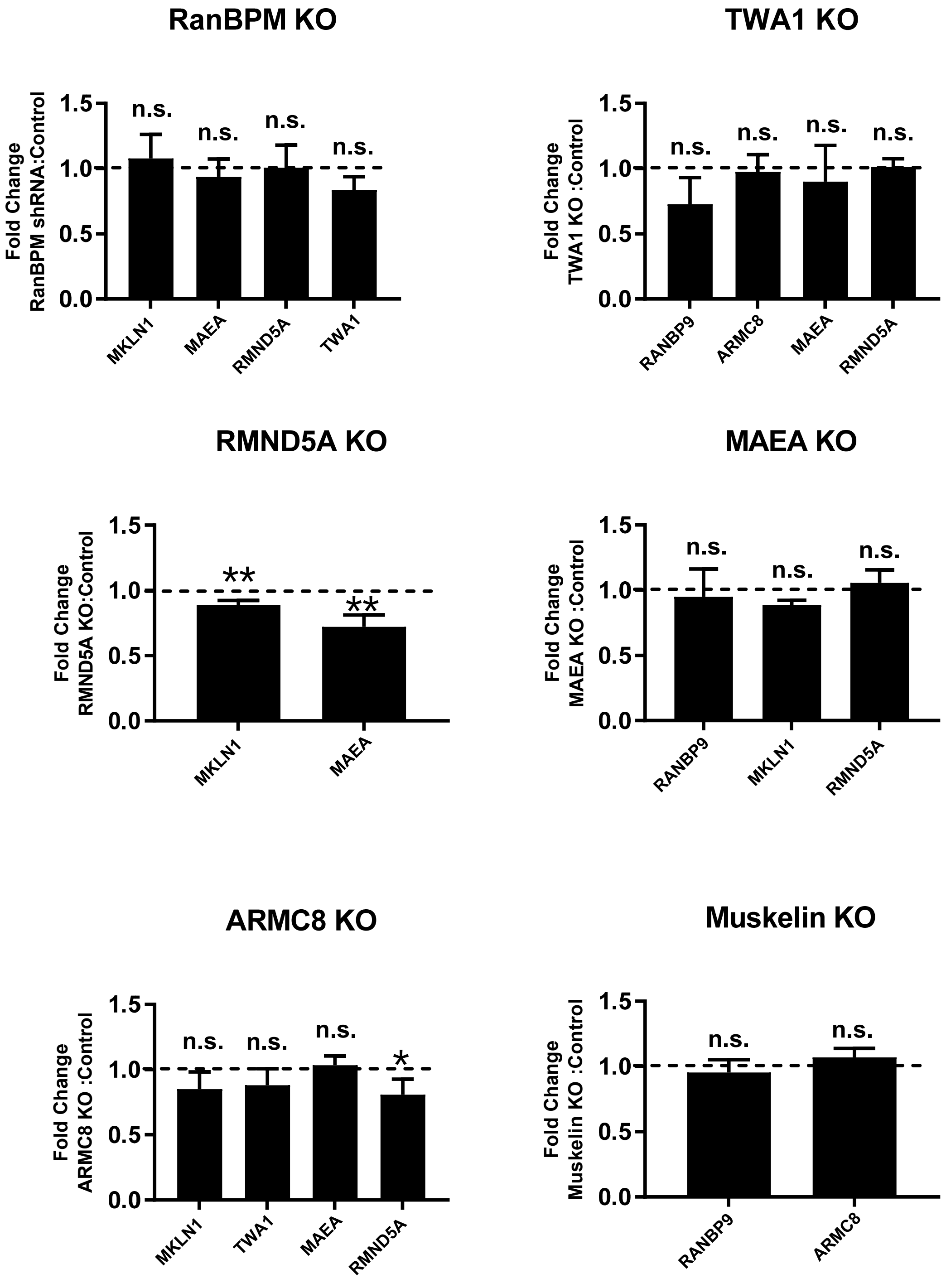

Supplementary Figure 3. Confirmation of changes of CTLH member protein expression in knockout cells or RanBPM shRNA cells by alternative knockdown/knockout strategies or rescue experiments. Related to figure 3.

Western blot analyses of whole cells extracts of: **a.** HEK293 RanBPM knockout cells. **b.** HEK293 cells transiently transfected with control or TWA1 siRNA. **c.** HEK293 cells transiently transfected with control or RMND5A siRNA. **d.** HEK293 cells transiently transfected with control or muskelin siRNA. **e.** HEK293 MAEA knockout cells untransfected or transiently transfected with FLAG-MAEA (as indicated at the top).

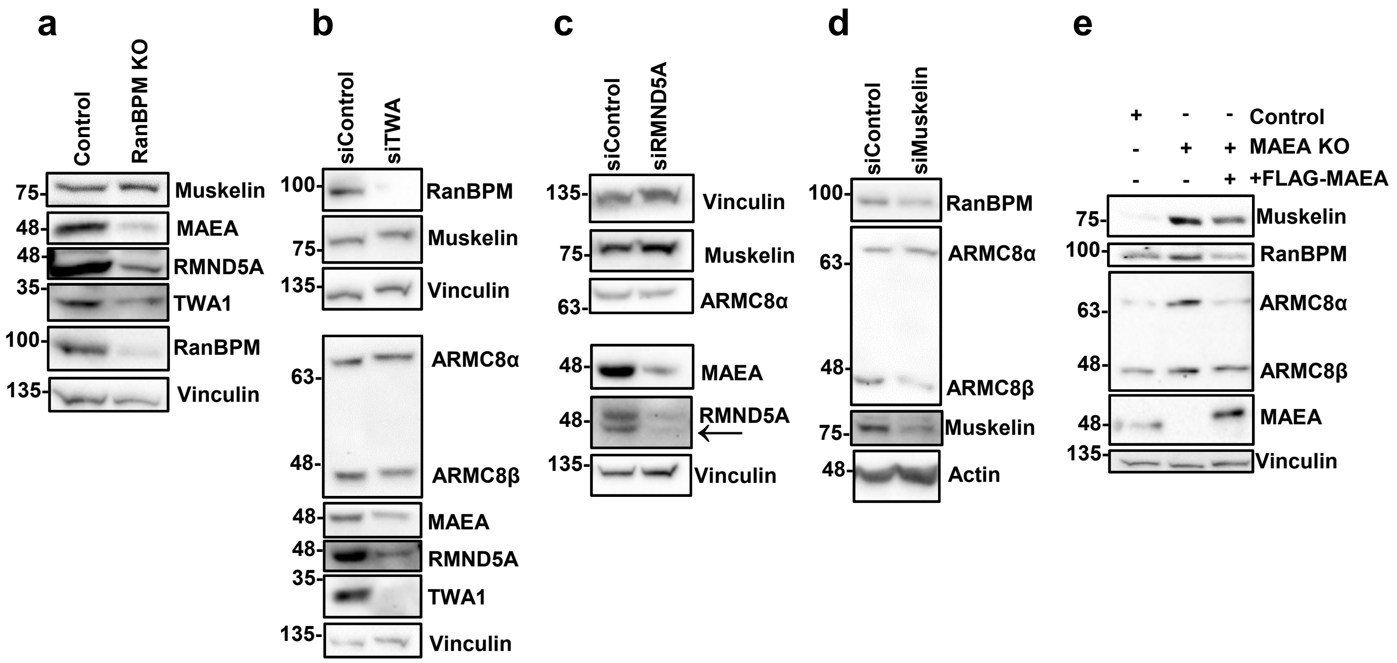

Supplementary Figure 4. CTLH complex is not compromised in RMND5A KO cells. WT or RMND5A KO HEK293 whole cell extracts were incubated with a RanBPM antibody and immunoprecipitated. Immunoprecipitates were analyzed by Western blot with the indicated antibodies. IgG was used as a negative control.

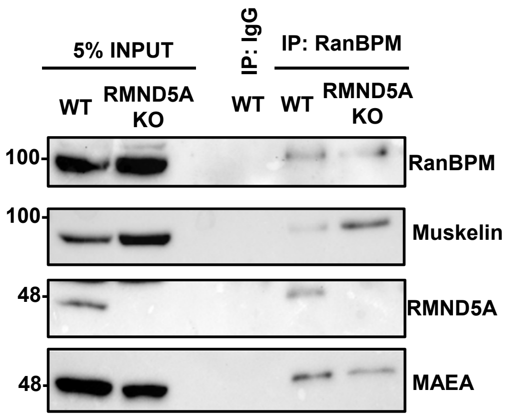

Supplementary Figure 5. Related to Fig. 6a. a) Uncropped image for Fig. 6a.

b) Negative control assay with GST empty vector.

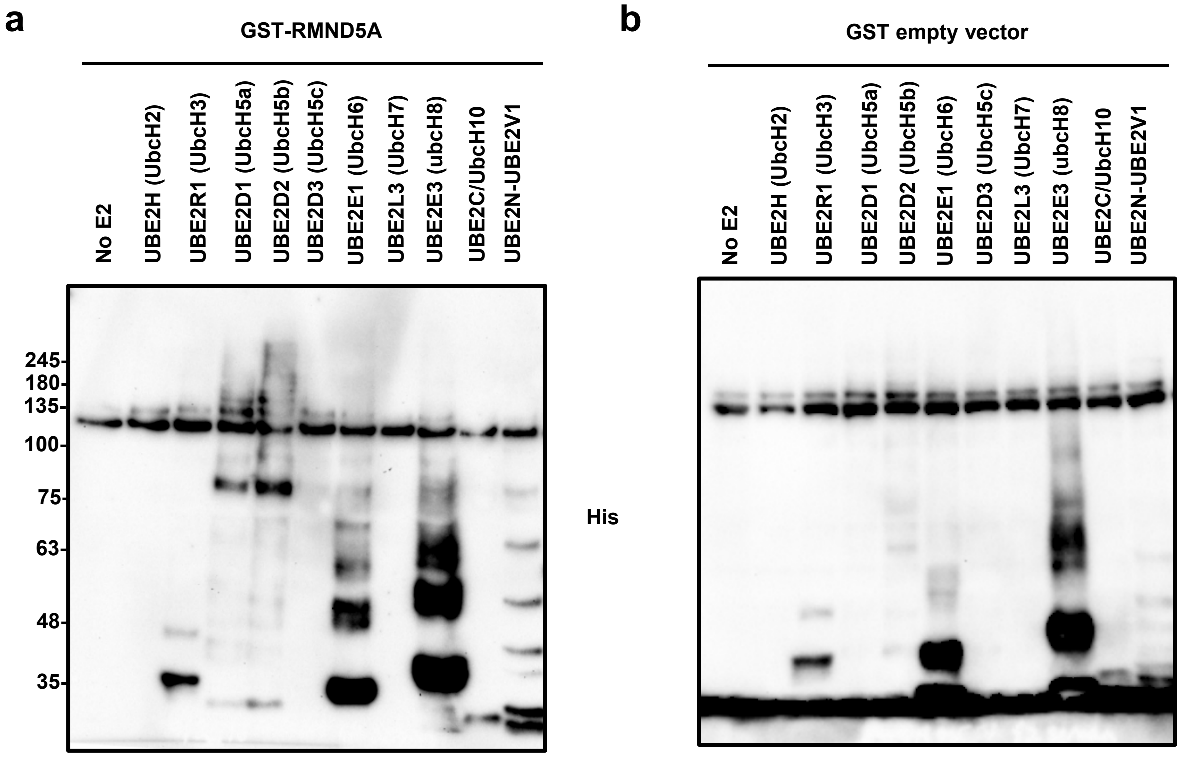

Supplementary Figure 6. Related to Fig. 6c. Ubiquitin peptides with GlyGly sites on K48 or K63 as quantified by MaxQuant for GST empty vector or GST-RMND5A ubiquitination assays.

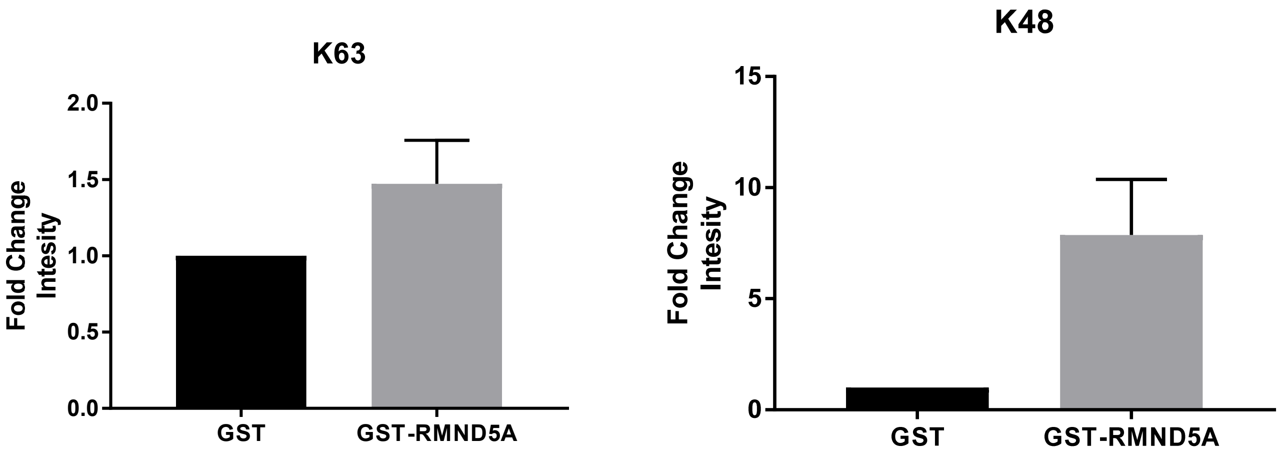

Supplementary File - Uncropped Blots

accompanying the manuscript entitled

The mammalian CTLH complex is an E3 ubiquitin ligase that targets its subunit muskelin for degradation

Matthew E.R. Maitland[1, 2, 3], Gabriel Onea[1, 2], Christopher A. Chiasson[1, 2], Xu Wang[1], Jun Ma[2,3], Sarah E. Moor[1, 2], Kathryn R. Barber[2], Gilles A. Lajoie[2, 3], Gary S. Shaw[2], and Caroline Schild-Poulter[1, 2, *]

[1]Robarts Research Institute, Schulich School of Medicine & Dentistry, The University of Western Ontario, London, Ontario, Canada.

[2]Department of Biochemistry, Schulich School of Medicine & Dentistry, The University of Western Ontario, London, Ontario, Canada.

[3]Don Rix Protein Identification Facility, Schulich School of Medicine & Dentistry, The University of Western Ontario, London, Ontario, Canada

[*]Correspondence: cschild-poulter@robarts.ca.

Uncropped immunoblots of Figure 1b.

RanBPM:

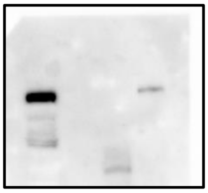

WDR26:

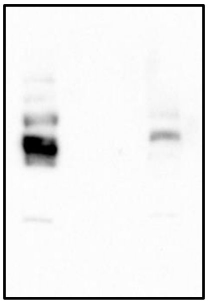

Muskelin:

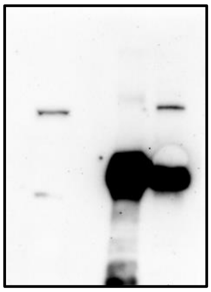

ARMC8:

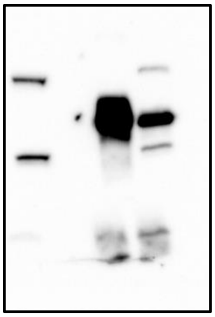

RMND5A:

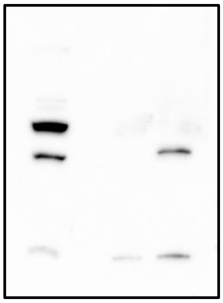

MAEA:

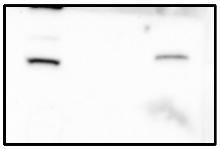

Uncropped immunoblots of Figure 1c.

FLAG:

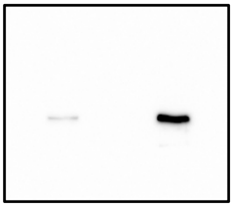

Muskelin:

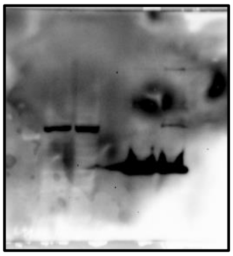

RanBPM:

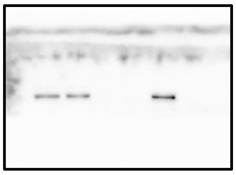

RMND5A:

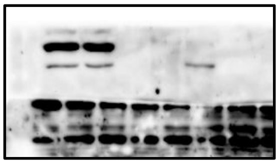

Different membrane and antibody

MAEA:

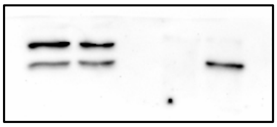

Uncropped immunoblots of Figure 1d.

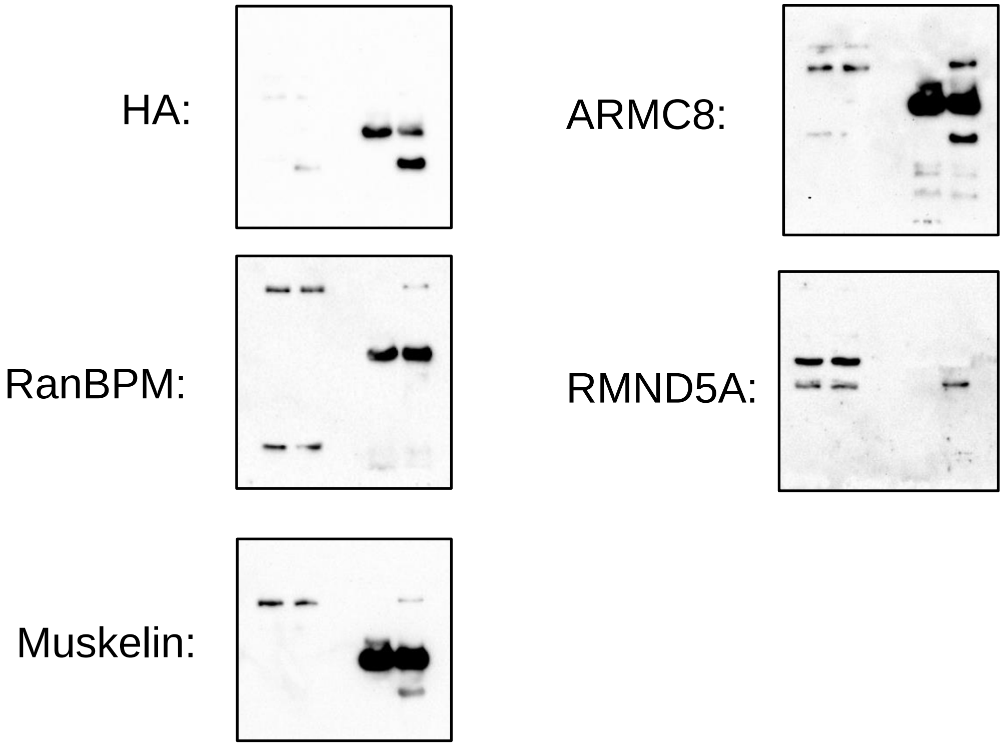

Uncropped immunoblots of Figure 3a. Note: Membrane was cut into strips prior to hybridization.

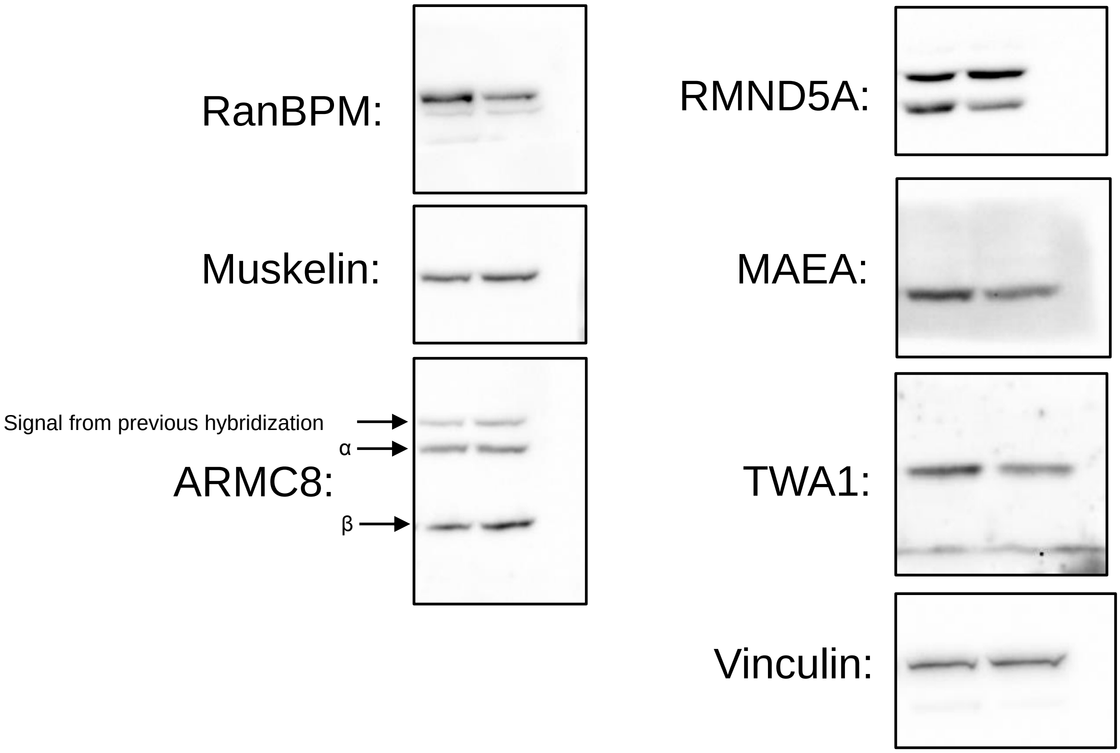

Uncropped immunoblots of Figure 3b. Note: Membrane was cut into strips prior to hybridization.

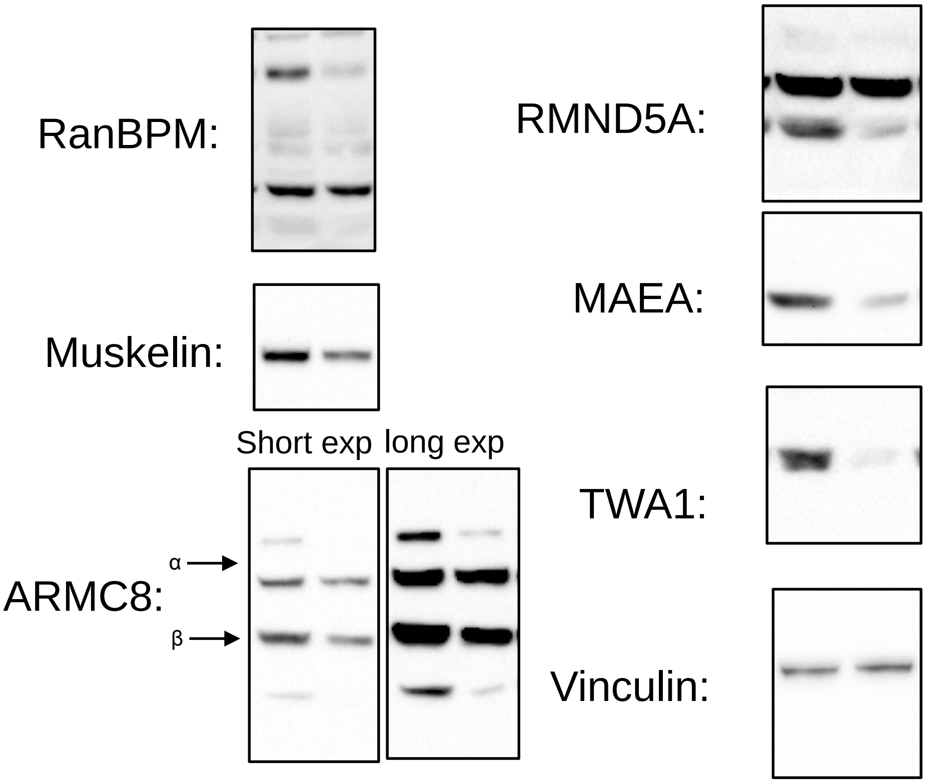

Uncropped immunoblots of Figure 3c. Note: Membrane was cut into strips prior to hybridization.

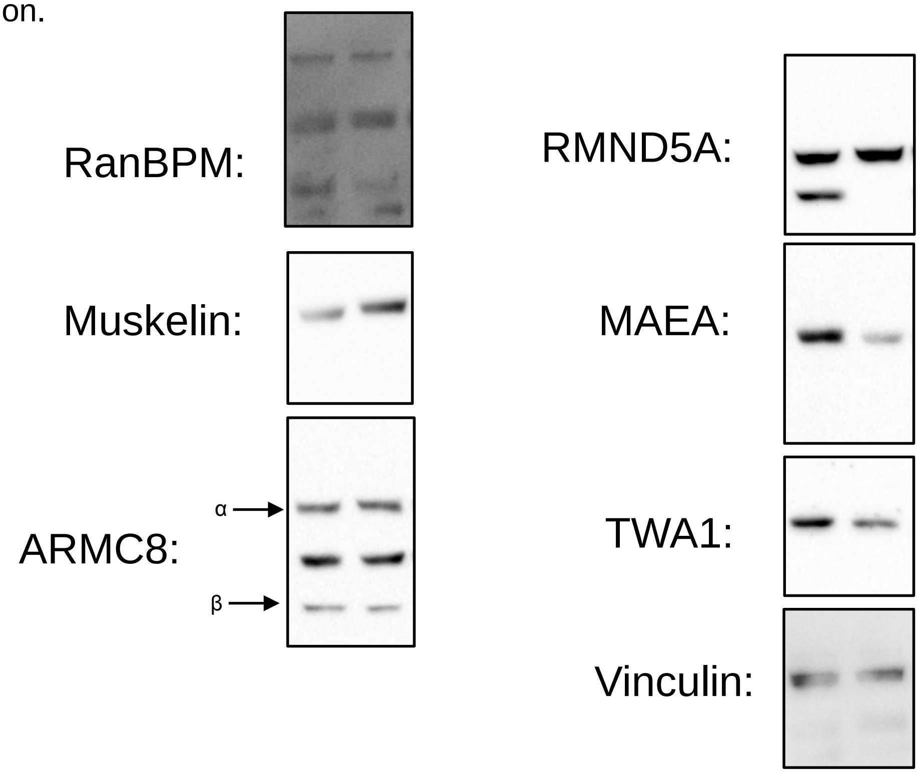

Uncropped immunoblots of Figure 3d. Note: Membrane was cut into strips prior to hybridization.

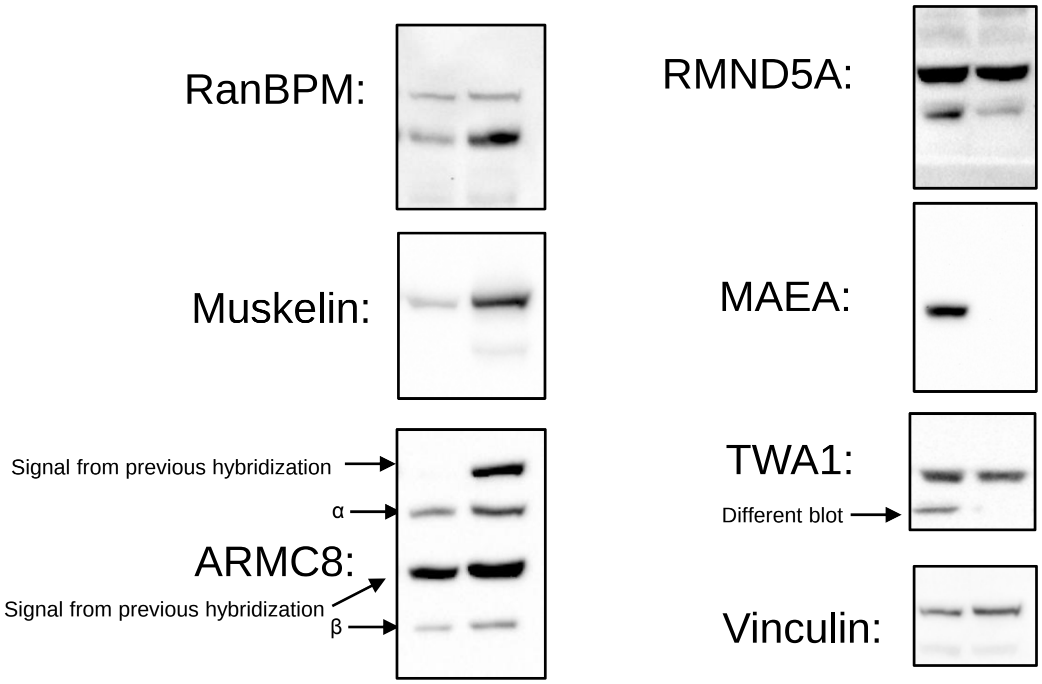

Uncropped immunoblots of Figure 3e. Note: Membrane was cut into strips prior to hybridization.

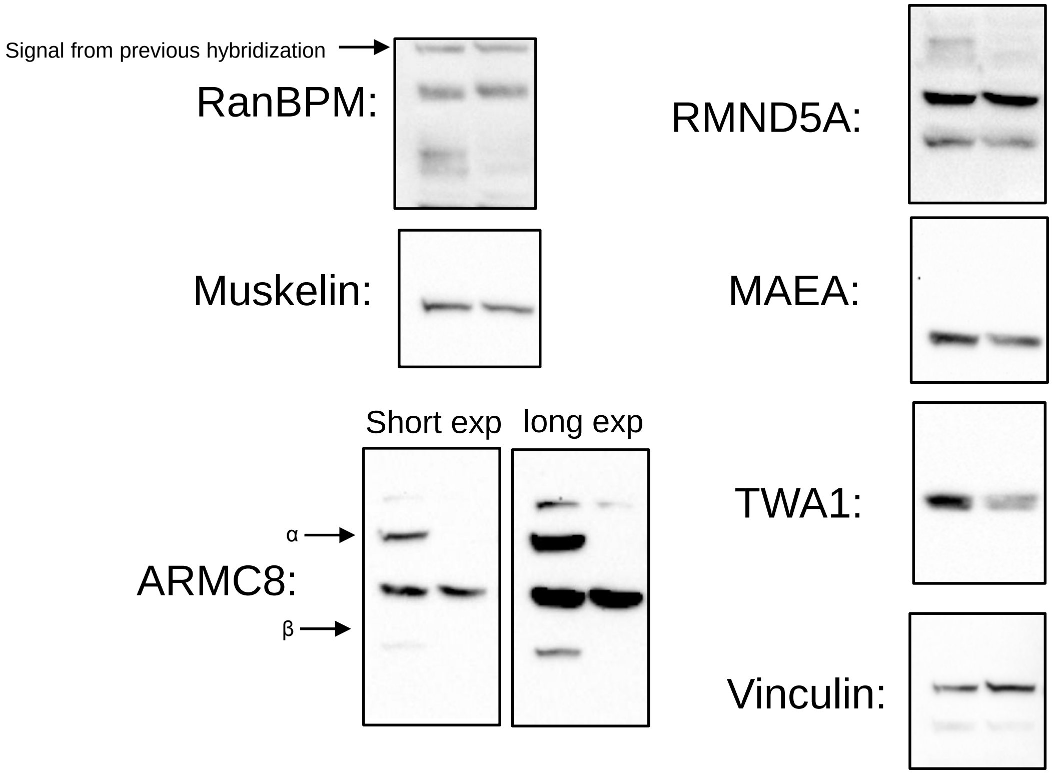

Uncropped immunoblots of Figure 3f. Note: Membrane was cut into strips prior to hybridization. Same extracts, two different gels (Gel #1 = RanBPM, Muskelin, Vinculin; Gel #2 = RMND5A. MAEA, TWA, ARMC8, Vinculin).

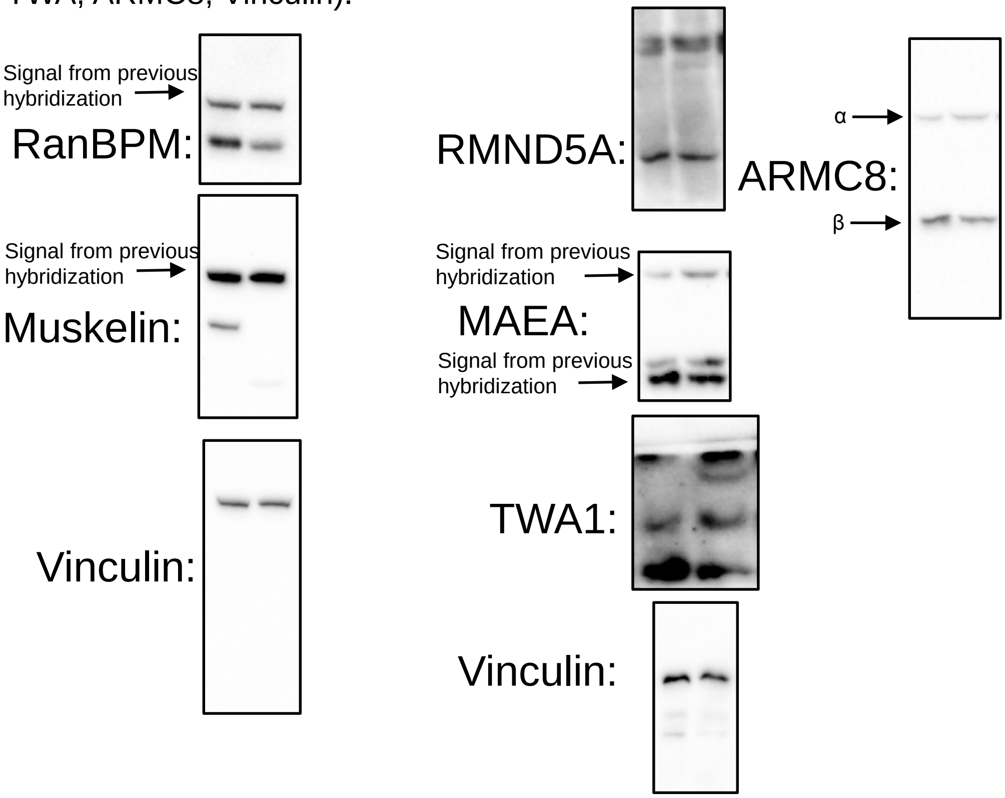

Uncropped immunoblots of Figure 4a.

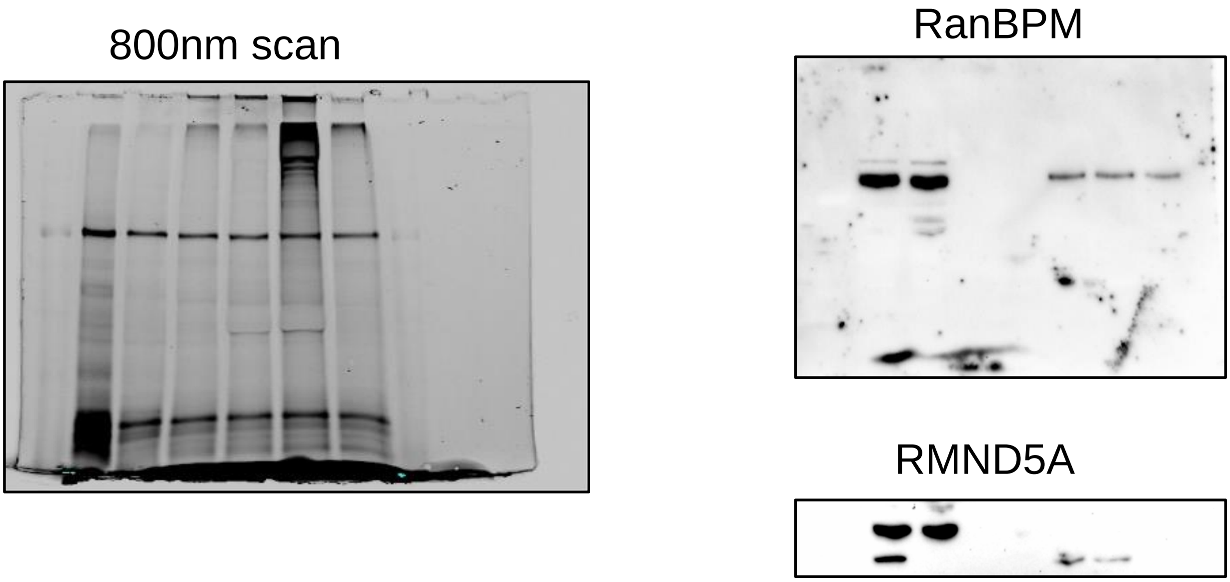

Uncropped scan and immunoblots of Figure 4b.

800nm scan

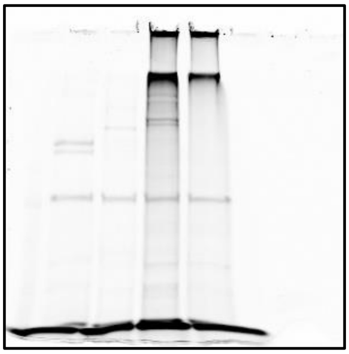

MAEA

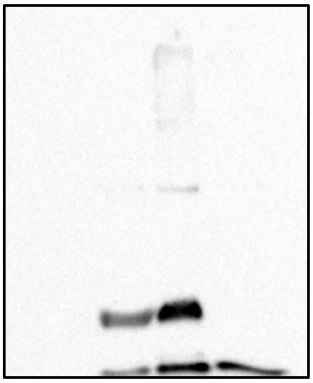

RanBPM

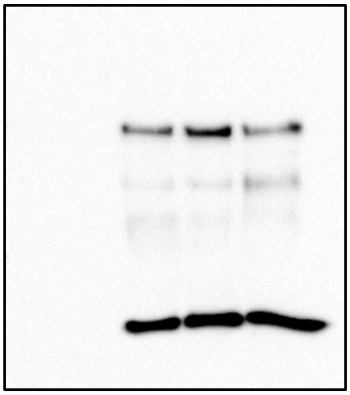

RMND5A

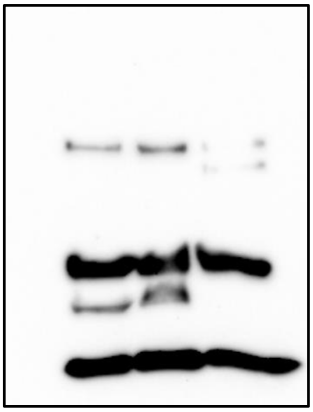

Uncropped scan immunoblot of Figure 6b.

800nm scan

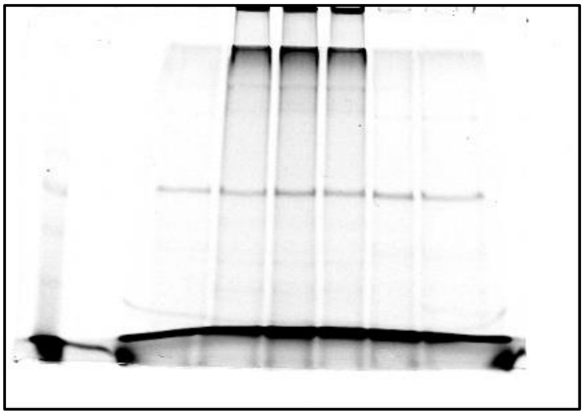

RanBPM

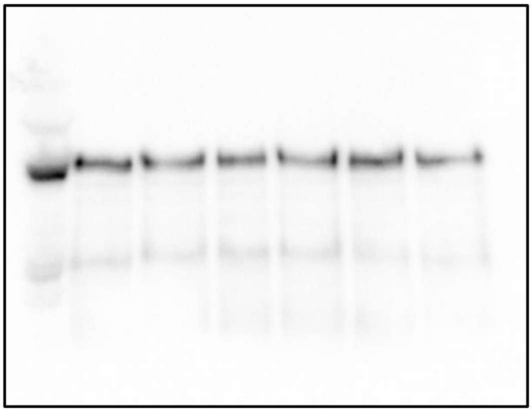

Uncropped immunoblots of Figure 5a.

Anti His

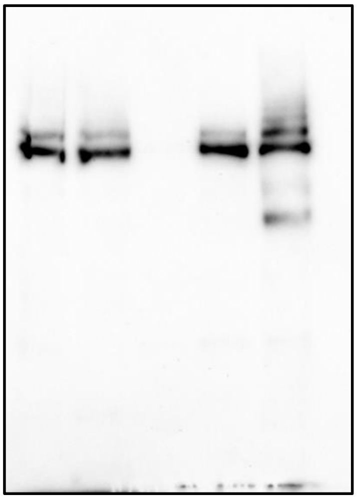

Anti His merged with ladder

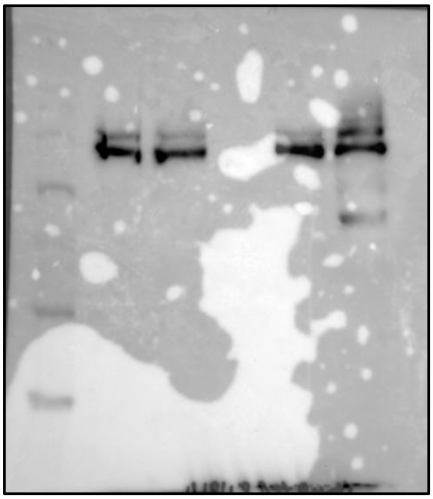

Anti RMND5A merged with ladder

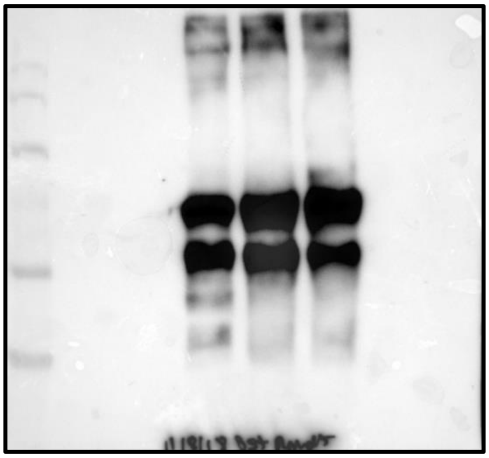

Uncropped immunoblots of Figure 5b.

Anti His

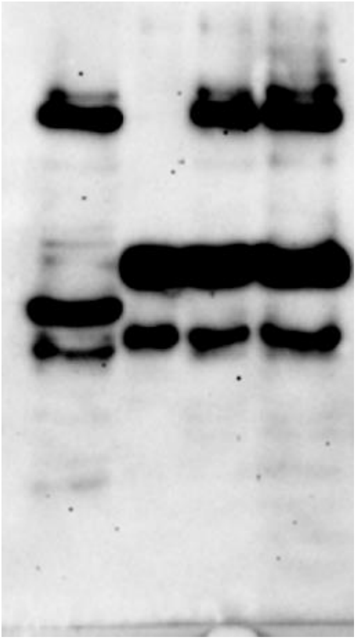

Anti His merged with ladder

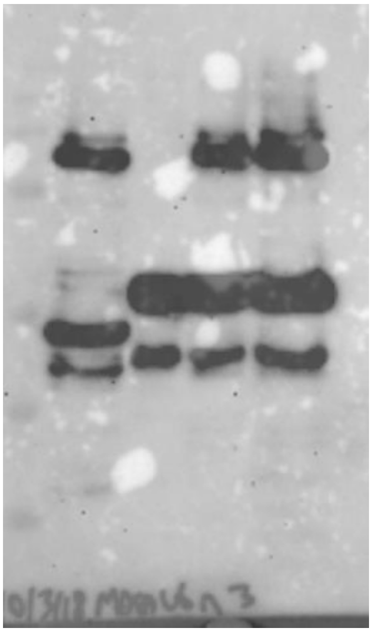

Anti MAEA merged with ladder

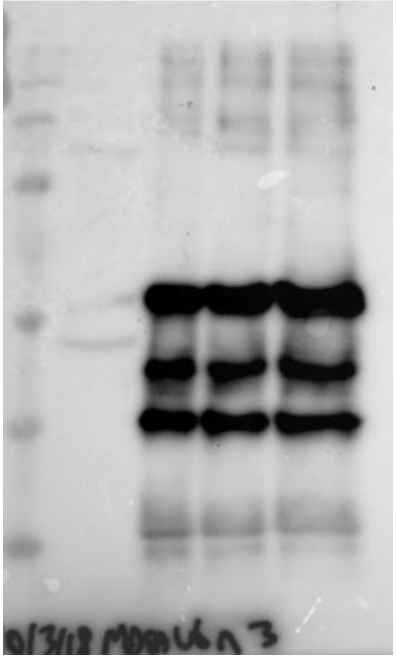

Uncropped immunoblots of Figure 7a. Note: Membrane was cut into strips prior to hybridization.

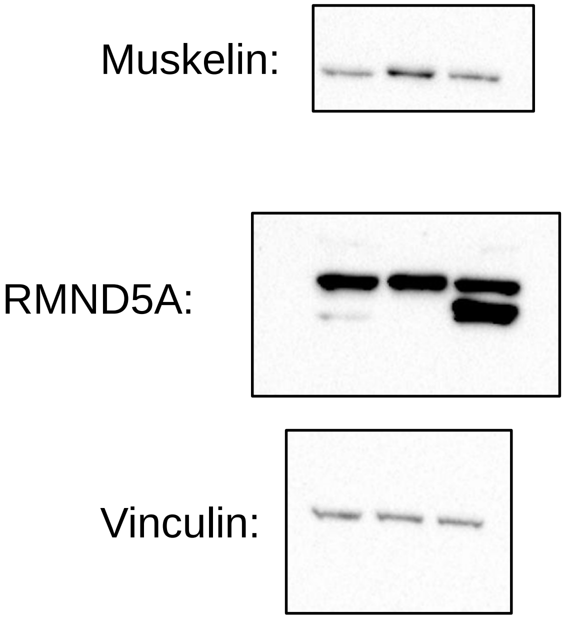

Uncropped immunoblots of Figure 7b. Note: Membrane was cut into strips prior to hybridization.

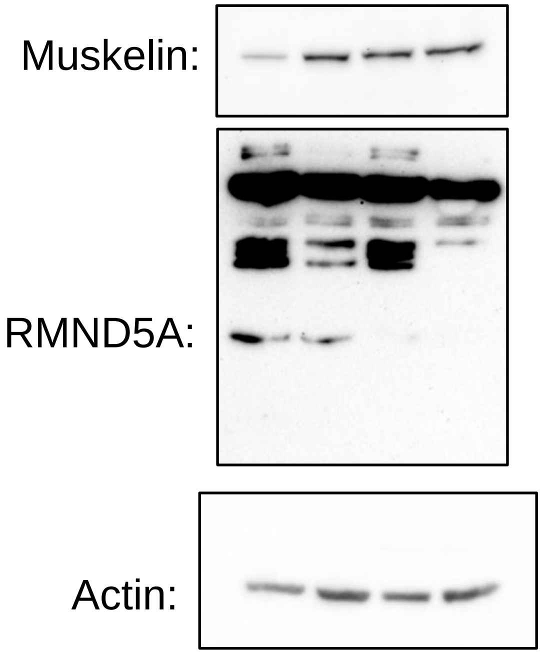

Uncropped immunoblots of Figure 7c. Note: Membrane was cut into strips prior to hybridization.

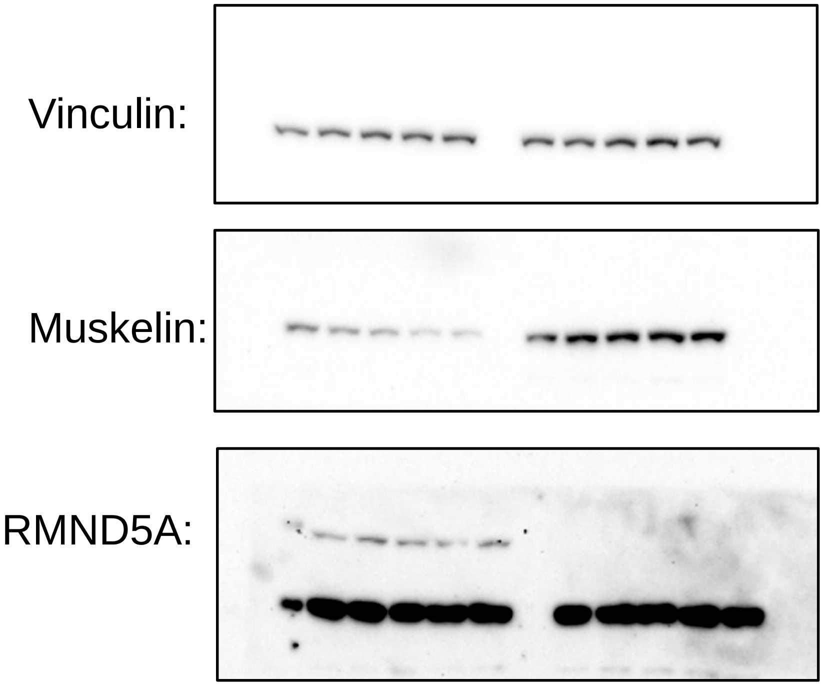

Uncropped immunoblots of Figure 7c. Note: IP eluant was split in half and run on two separate gels to avoid overlapping signal.

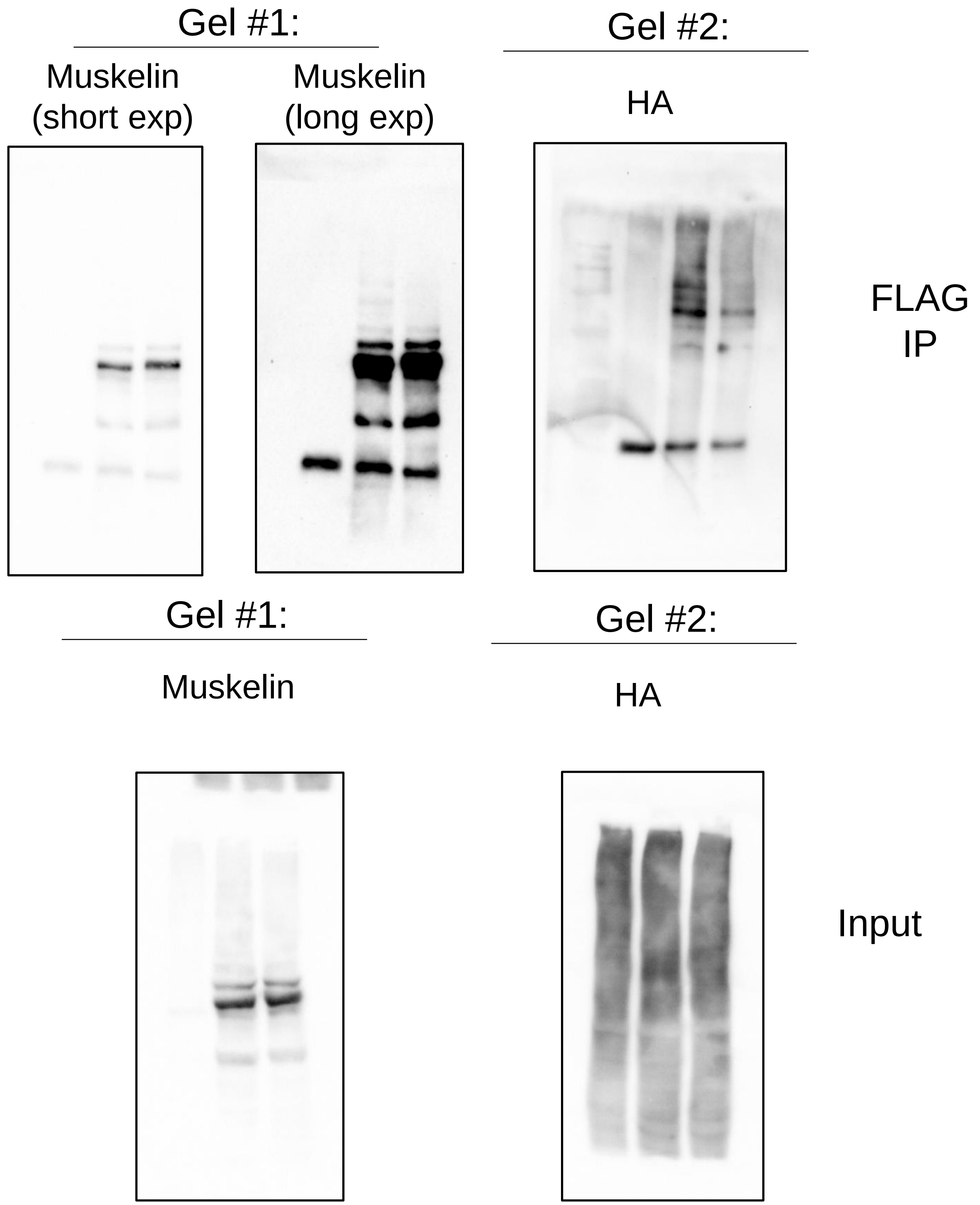

Uncropped immunoblots of Figure 7d.

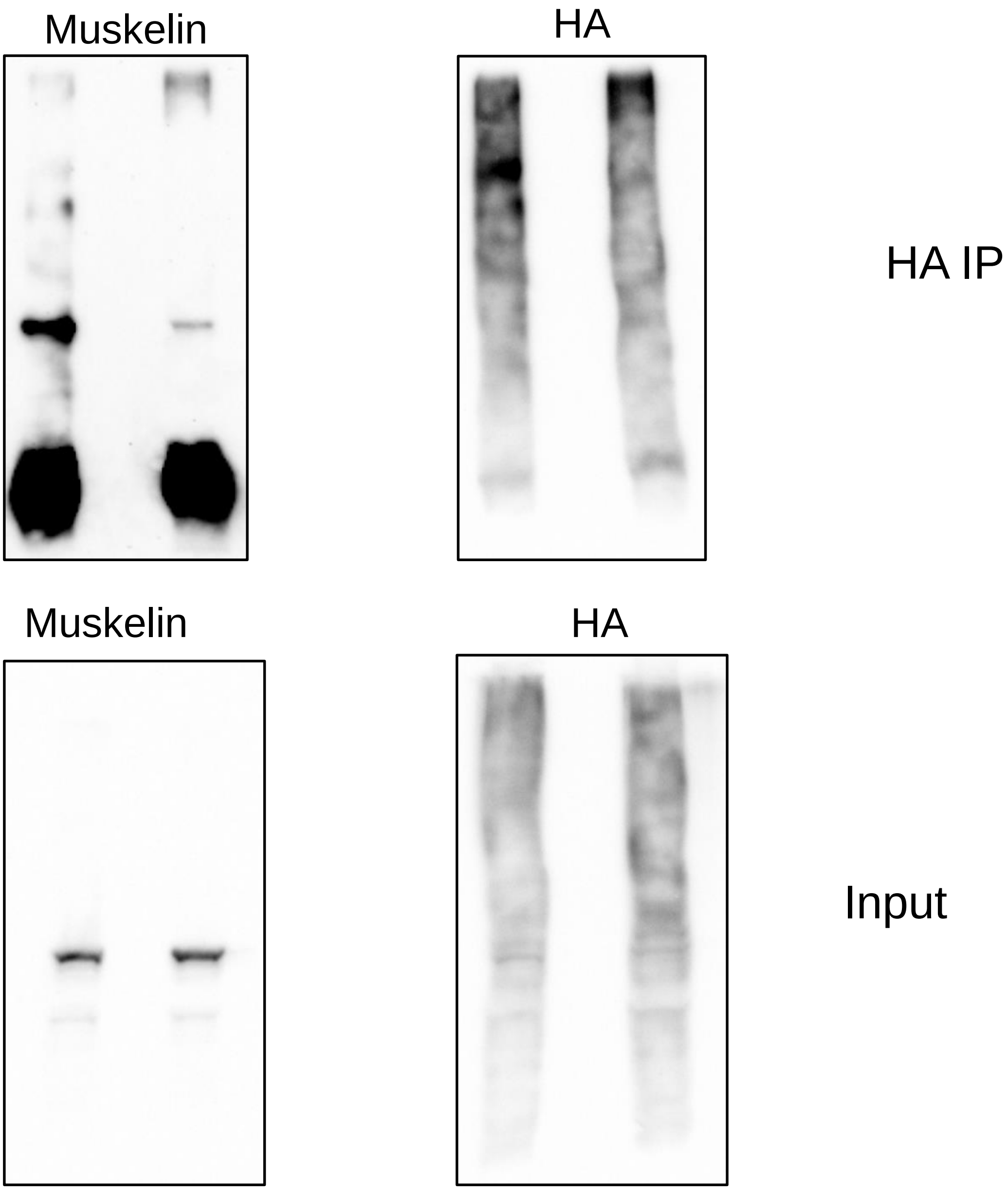

Uncropped immunoblots of Supplemental Figure 1.

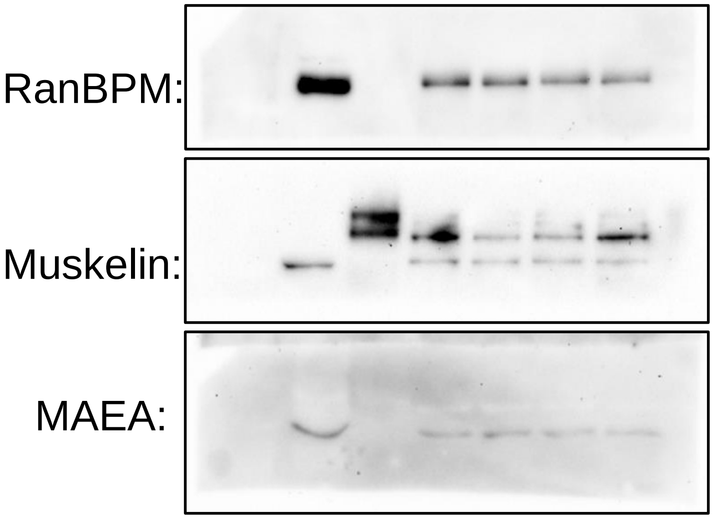

Uncropped immunoblots of Supplemental Figure 3.

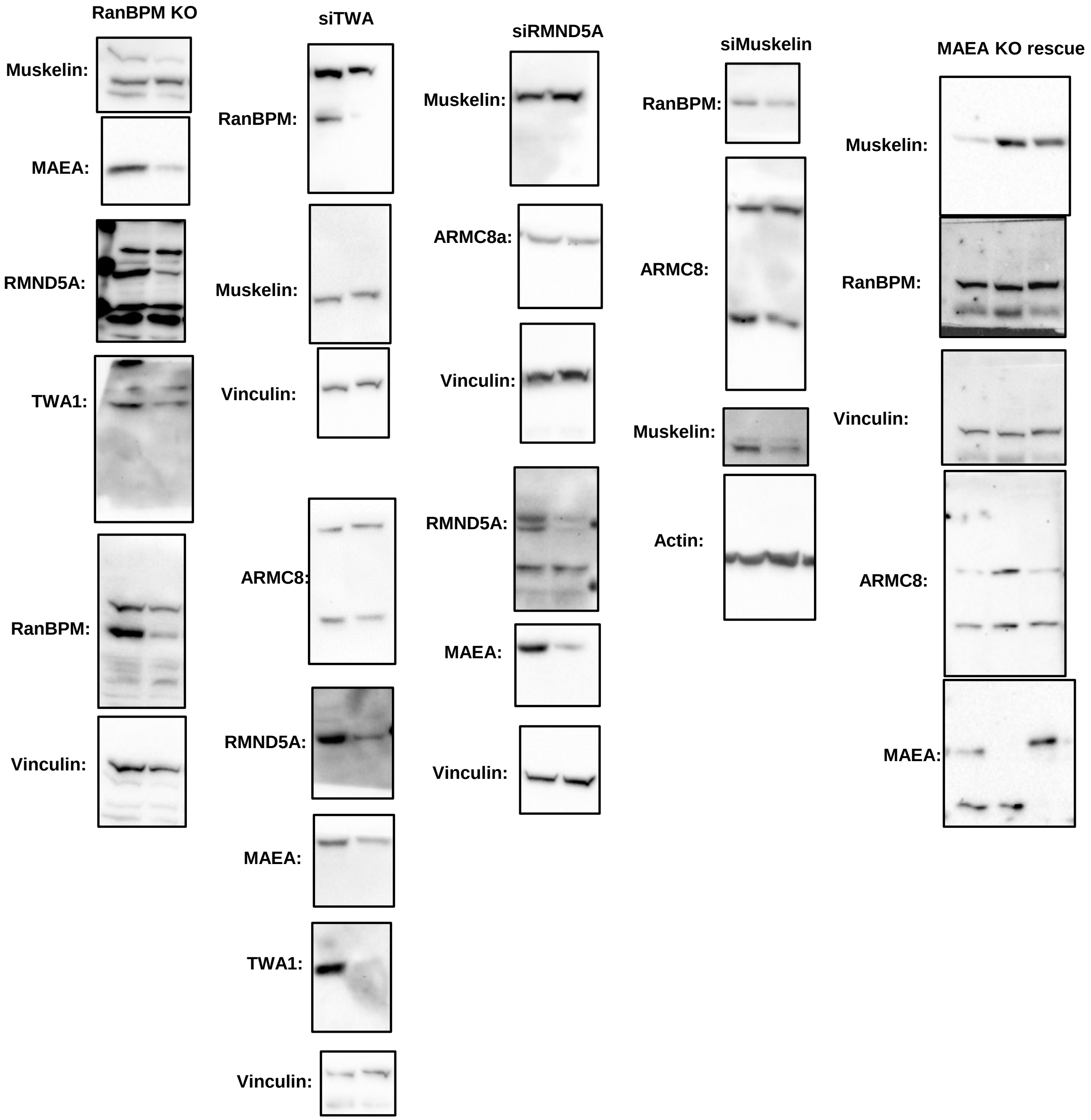

Supplement: Supplementary file 1 — Supplementary Figures 1 to 6 and Uncropped Blots [file 41598_2019_46279_MOESM1_ESM.pdf]
